# Genome position and gene amplification

**DOI:** 10.1186/gb-2007-8-6-r120

**Published:** 2007-06-21

**Authors:** Pavla Gajduskova, Antoine M Snijders, Serena Kwek, Ritu Roydasgupta, Jane Fridlyand, Taku Tokuyasu, Daniel Pinkel, Donna G Albertson

**Affiliations:** 1 Cancer Research Institute, University of California San Francisco, San Francisco, CA 94143-0808, USA; 2 Comprehensive Cancer Center, University of California San Francisco, San Francisco, CA 94143-0808, USA; 3 Department of Epidemiology and Biostatistics, University of California San Francisco, San Francisco, CA 94143-0808, USA; 4 Department of Laboratory Medicine, University of California San Francisco, San Francisco, CA 94143-0808, USA; 5 Institute of Biophysics, Academy of Sciences of the Czech Republic, Královopolská, Brno, 612 65, Czech Republic

## Abstract

**Background:**

Amplifications, regions of focal high-level copy number change, lead to overexpression of oncogenes or drug resistance genes in tumors. Their presence is often associated with poor prognosis; however, the use of amplification as a mechanism for overexpression of a particular gene in tumors varies. To investigate the influence of genome position on propensity to amplify, we integrated a mutant form of the gene encoding dihydrofolate reductase into different positions in the human genome, challenged cells with methotrexate and then studied the genomic alterations arising in drug resistant cells.

**Results:**

We observed site-specific differences in methotrexate sensitivity, amplicon organization and amplification frequency. One site was uniquely associated with a significantly enhanced propensity to amplify and recurrent amplicon boundaries, possibly implicating a rare folate-sensitive fragile site in initiating amplification. Hierarchical clustering of gene expression patterns and subsequent gene enrichment analysis revealed two clusters differing significantly in expression of MYC target genes independent of integration site.

**Conclusion:**

These studies suggest that genome context together with the particular challenges to genome stability experienced during the progression to cancer contribute to the propensity to amplify a specific oncogene or drug resistance gene, whereas the overall functional response to drug (or other) challenge may be independent of the genomic location of an oncogene.

## Abstract 2

Genomic analyses of human cells expressing dihydrofolate reductase provide insight into the effects of genome position on the propensity for a drug-resistance gene to amplify in human cells.

## Background

Genetic instability resulting in chromosomal level alterations is frequent in solid tumors, which display a wide variety of types and frequencies of these aberrations. Amplifications, regions of focal high level copy number change, are likely to represent aberrations continuously under selection during tumor growth, since amplified DNA is unstable [1-4] and would otherwise disappear. They often harbor known oncogenes and thus are useful for identifying genes or pathways that foster tumor development. For *ERBB2*, amplification is the predominant method of its up-regulation and is the basis for FISH-based tests evaluating the ERBB2 status of breast tumors. On the other hand, change in DNA copy number is only one way to alter expression of a gene and expression of other oncogenes is much less tightly linked to DNA copy number or amplification. Other mechanisms for deregulation may be post-transcriptional, post-translational or involve alteration in expression of upstream genes. Tumor subtypes may also be distinguished by their propensity to amplify oncogenes, suggesting that the particular types of genomic instability present in a tumor are important determinants of how expression of an oncogene might be altered. Moreover, amplification is often associated with poor prognosis.

Amplification is a reiterative process, in which multiple copies of a genome region are accumulated. Studies in model systems indicate that amplification requires a DNA double strand break and progression through the cell cycle with this damaged DNA [5-9]. A role for genome context in promoting amplification has also been suggested, since introduction of a selectable gene into different genome positions in hamster and yeast cells resulted in site-dependent frequencies of resistant colonies following drug challenge [10,11]. Particular genome sequences prone to breakage have also been shown to set the boundaries of amplicons in rodent cells [6,12], further suggesting that genome position influences the propensity to amplify.

Common chromosomal fragile sites, of which there are approximately 90 in the human genome, have received the most attention as sites likely to promote amplification. Expression of fragile sites can be induced in cells in culture under conditions of replication stress and are visualized as gaps on metaphase chromosomes. Fragile sites can be divided into common sites (CFS), which are seen in all individuals and rare sites (RFS), which appear only in certain individuals. The sites are further distinguished by agents used to induce expression, which include aphidicolin, bromo-deoxyuridine (BrdU), 5-azacytidine and distamycin A. Folate stress caused by methotrexate exposure also induces a group of rare fragile sites. A small number of CFS have been molecularly identified and found to vary from hundreds of kilobases to over one megabase in size, to have some unusual sequence properties, but not to be conserved in sequence. Often they contain very large genes and are sites of viral integration in certain cancers [13]. Evidence supporting a role for fragile sites in promoting amplification in human cancer is provided by the *MET *oncogene, which is amplified in esophageal adenocarcinoma. The gene lies within FRA7G, and the amplicon boundaries in tumors also lie within this site [14]. Nevertheless, for many amplicons there is no obvious involvement of common chromosomal fragile sites.

Gene amplification has been studied *in vitro *in a variety of systems by selection for cells capable of growth in the presence of antimetabolites. To investigate the role of genome context on amplification in human cells, we chose methotrexate resistance as the model system, because clinical resistance to methotrexate targets a number of genes by a variety of mechanisms [15,16], thereby providing the opportunity to determine which types of aberration occur more frequently in different genetic backgrounds. We introduced a mutant copy of *DHFR*, which confers greater resistance to methotrexate than the endogenous wild-type *DHFR*, into random sites in the genome of chromosomally stable HCT116+chr3 cells and precisely determined the site of single copy integrations in the human genome sequence. We isolated colonies resistant to folate deprivation caused by methotrexate and characterized these cells with respect to site specific response to drug challenge. We used array comparative genomic hybridization (CGH) to identify and classify the types of genomic alterations in the drug resistant cells, fluorescent *in situ *hybridization (FISH) to study the organization and mechanism of amplicon formation, and expression profiling to investigate the functional consequences of amplification. These studies found site specific differences in the sensitivity to methotrexate, organization of amplicons and propensity to amplify. On the other hand, gene expression patterns of drug resistant cells were independent of integration site, with two major clusters revealed by hierarchical clustering of the expression profiles. The clusters differed significantly in expression of *MYC *target genes. Translated to human disease, these studies suggest that genome context together with the particular challenges to genome stability experienced during the progression to cancer contribute to the propensity to amplify a specific oncogene, whereas the overall functional response to drug (or other) challenge may be independent of the genomic location of an oncogene.

## Results

### Characteristics of clones with DHFR* integration

To study formation and structure of amplicons, we took advantage of the fact that resistance to methotrexate can be accomplished by a number of mechanisms, including copy number gain or amplification of *DHFR *and loss or down regulation of the folate transporter, *SLC19A1 *on chromosome 21 [15,16]. We have shown previously that HCT116+chr3 cells have stable karyotypes and that methotrexate resistant HCT116+chr3 cells frequently amplify *DHFR *on chromosome 5q [17]. The HCT116+chr3 cells are a variant of the mismatch repair deficient colorectal carcinoma cell line, HCT116; however, they are mismatch repair proficient due to the wild-type copy of *MLH1 *provided by an extra copy of chromosome 3p and proximal 3q [17,18]. Because these cells carry two wild-type copies of *DHFR*, we introduced a mutant form of *DHFR *(L22F), which confers greater resistance to methotrexate than the wild-type (endogenous) gene, into HCT116+chr3 cells by retroviral infection. The *DHFR *(L22F) variant also was fused to the gene encoding enhanced green fluorescence protein (EGFP) and is referred to here as DHFR*. We isolated 56 independent clones containing DHFR* at different positions in the genome and identified genome sequences flanking the integration site of DHFR* using inverse PCR (Additional data file 1). For further analysis, we selected only clones that were considered to have a single insertion of DHFR* by inverse PCR (13 independent insertion sites, Table 1).

**Table 1:** Thirteen DHFR* insertion site clones and their response to methotrexate

Name	Chr.	Chr. band	Sequence^1 ^(bp)	Exp.^2^	IC-50^3 ^(nM)	MTX^4 ^(nM)	Expression^5 ^(%)	No. of colonies^6^	Colonies screened^7^	DHFR* amplicon^8^	DHFR* gain^9^
1M-34	9	q34.3	137,580,684	→	43	120	4,172	26.5 (8.50)	10	4	0
1M-39	13	q32.1	96,707,451	←	22	65	3,835	0.5 (1.00)	0	0	0
1M-42	8	q22.3	102,162,871	←	21	75	2,660	5.5 (3.25)	15	13	2
1M-43	11	q13.1	65,526,684	→	22	65	1,828	13 (10.50)	9	0	3
1M-45	11	q23.3	118,293,388	←	70	200	5,329	2.5 (3.00)	8	1	6
1M-57	3	q27.1	185,207,363	→	15	75	2,047	3.5 (3.75)	12	3	8
1M-67	1	p34.3	39,533,783	→	31	90	2,306	0.5 (1.00)	3	1	0
1M-72	2	q35	217,190,120	←	16	65	5,077	1.0 (2.00)	2	1	1
1M-73	12	p13.2	12,767,229	←	20	65	3,116	0.5 (1.00)	1	0	0
1M-75	22	q12.2	28,977,607	→	32	90	3,307	25.5 (12.75)	3	1	1
1M-83	19	q13.2	45,618,743	→	14	65	756	1.0 (1.75)	6	0	1
1M-84	17	q12	34,161,068	→	23	75	3,934	11.0 (11.00)	4	0	0
1M-89	19	q13.33	53,682,371	→	25	75	4,288	2.0 (1.00)	9	3	0

^1^Position in the human genome sequence (UCSC Genome Browser, May 2004 freeze). ^2^Exp., Direction of DHFR* integration in the genome; arrow to the right indicates that the DHFR* coding sequence is integrated in the direction from the p-arm to the q-arm of the chromosome. ^3^Methotrexate concentration that causes 50% inhibition of the cell growth after 6 days. ^4^Methotrexate (MTX) concentration used for selection of resistant colonies. ^5^Expression of DHFR* measured indirectly by quantitative RT-PCR (EGFP expression normalized to *GUSB *expression, 2^-(dCt) ^× 100). ^6^Median number (and interquartile range in parentheses) of resistant colonies per 10 cm plate after 28 days of methotrexate treatment. ^7^Number of independent resistant colonies screened by array CGH. ^8^Resistant colonies with amplification of the DHFR* integration region. ^9^Resistant colonies with low level copy number gain of the DHFR* integration region. Chr., chromosome.

**Figure 1:**
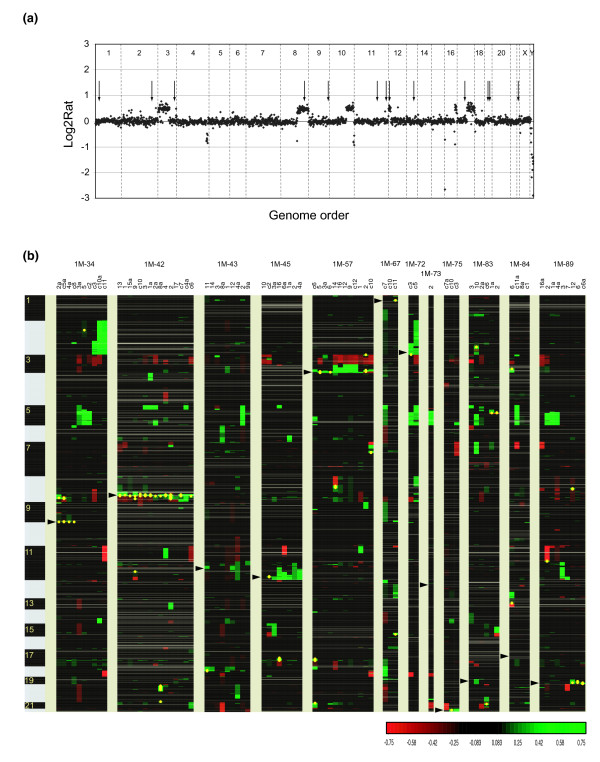
Parental DHFR* integration sites and copy number aberrations in methotrexate resistant colonies. **(a) **Copy number profile of cell line HCT116+chr3 and positions of 13 DHFR* integrations. This near-diploid cell line is characterized by partial chromosomal gains on chromosomes 3, 8, 10, 12, 16, losses on chromosomes 4, 16 and 10 and homozygous deletion on chromosome 16. Shown are the log_2 _ratios on BAC clones ordered according to genome position (UCSC Genome Browser, May 2004 freeze). Arrows indicate the positions of integration of one copy of DHFR* as mapped by inverse PCR to the human genome sequence. **(b) **Heatmap representation of copy number changes detected by array CGH in 82 methotrexate resistant colonies from 12 different insertion sites. Each column represents one resistant colony. Resistant colonies from each DHFR* integration site were clustered according to their copy number changes. Positions of the insertion sites are indicated by the arrowheads. Individual BAC clones are shown as rows and ordered according to their genome position (UCSC Genome Browser, May 2004 freeze). Copy number losses are indicated in red, gains in green and amplifications as yellow dots.

The individual insertion site clones were further characterized with respect to genome copy number profiles and expression of DHFR*. All clones with the exception of two (1M-39 and 1M-43) showed the same chromosomal changes by array CGH as HCT116+chr3 cells (Figure 1a). Clone 1M-39 gained part of the q-arm of chromosome 4 (between RP11-18D7 and the q-telomere). Clone 1M-43 contained one additional copy of chromosome 22 and had lost the q-arm of chromosome 18. We confirmed DHFR* expression in all clones by measuring the expression level of the fused EGFP portion of the gene using quantitative RT-PCR. Expression levels, as percentage of *GUSB *expression, showed an approximately seven-fold variation (Table 1).

### Frequency of DHFR* amplification at different genomic sites

Initially we measured the sensitivity of each clone to methotrexate by determining the methotrexate concentration that causes 50% reduction in cell number after six days exposure to varying concentrations of the drug (IC-50). The HCT116+chr3 cells showed the greatest sensitivity to the drug (IC-50 = ~7.6 nM). Clones with DHFR* showed 1.9- to 9.2-fold increase in methotrexate resistance (IC-50 ranged from 14.1 to 70.1 nM; Table 1). To select methotrexate resistant colonies, we exposed cells to a concentration of methotrexate that was three to four times the IC-50 for each integration site. We note that because *DHFR *is the target of methotrexate, exposure to the drug should inhibit synthesis of thymidylate and reduce levels of thymidine-based nucleotides. Such a reduction in nucleotide levels could cause DNA damage; however, the concentrations used here are not expected to do so [8]. Moreover, we determined that exposure of HCT116+chr3 cells to the range of concentrations used in these studies does not result in significant DNA damage as measured by the alkaline comet assay. The median number of resistant colonies obtained for each integration site is shown in Table 1.

Genomic copy number profiles were obtained for isolated resistant colonies that grew sufficiently well to be expanded to 5 × 10^6 ^cells (Figure 1; Additional data file 2). The retention of DHFR* at the original site of integration was confirmed in all resistant colonies using inverse PCR, as shown for untreated clone 1M-89 and its resistant colonies in Additional data file 1. Clones from different integration sites could be separated into four groups: The first group contained only one clone, 1M-39, which did not form any resistant colonies. The second group (1M-73 and 1M-84) formed resistant colonies that did not amplify DHFR*; however, partial gain of chromosome 5 and loss of chromosome 21 were among the copy number changes, suggesting that increased copies of the endogenous *DHFR *locus and loss of *SLC19A1 *contributed to resistance. Clones in the third group (1M-43 and 1M-83) showed low level copy number changes (partial or whole chromosome gains) of the region with DHFR* integration in at least one resistant colony. Finally, clones in the fourth group (1M-34, 1M-42, 1M-45, 1M-57, 1M-67 1M-72, 1M-75 and 1M-89) formed methotrexate resistant colonies with amplicons around the DHFR* integration site at varying frequencies (Table 1).

### Structure of amplicons and mechanisms of formation

**Figure 2:**
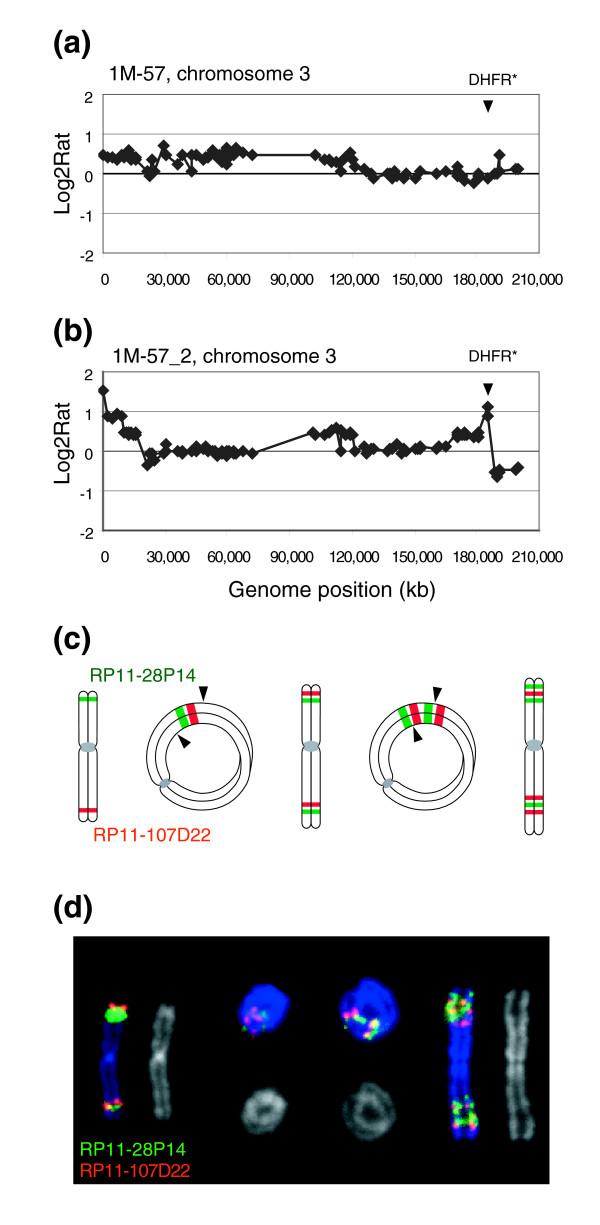
Mechanism of DHFR* amplification involving a ring chromosome intermediate. **(a) **Chromosome 3 copy number profile in untreated 1M-57 cells. Shown are the log_2 _ratios ordered according to position on the May 2004 freeze of the human genome sequence. The copy number gain extending from 3pter to RP11-233L3 reflects the presence of the two normal copies of chromosome 3 and the additional piece of chromosome 3 in HCT116+chr3 cells. The DHFR* insertion site is indicated by the arrowhead. **(b) **Chromosome 3 copy number profile in methotrexate resistant colony 1M-57_2. Two regions of amplification are evident at 3pter and near the distal end of 3q. Copy number losses include material from 3p and 3qter distal to DHFR*. **(c) **Proposed mechanism leading to amplification of the region around DHFR*. The ends of chromosome 3 fuse to create a ring chromosome with loss of material distal to DHFR* on 3q. Breakage at positions indicated by the arrowheads at anaphase results in the metacentric chromosome that now carries duplication of juxtaposed 3p and 3q sequences. The process can be repeated to generate additional copies of the 3p and 3q sequences. **(d) **FISH with RP11-107D22 (red), which contains sequences flanking the DHFR* integration site on 3q and RP11-28P14 (green), which maps to 3p. Shown are pseudocolor images showing hybridization signals on the chromosomes and the corresponding DAPI image (gray).

**Figure 3:**
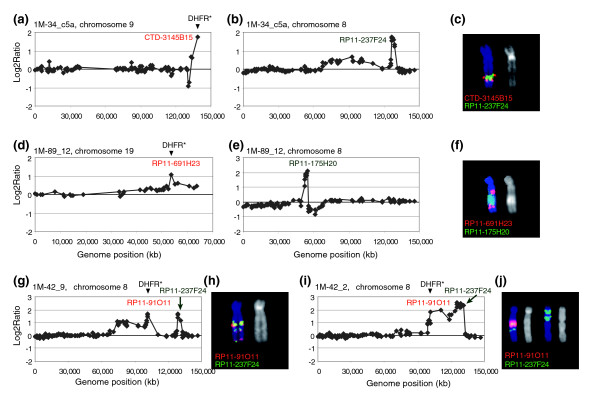
Amplicons formed by two genomic regions, initially located on the different chromosomes. Chromosome 8 and 9 copy number profiles showing amplification on **(a) **9q and **(b) **8q. **(c) **Organization of the amplicon. CTD-3145B15 (red) maps to the 9q telomere near the DHFR* insertion site and RP11-237F24 (green) to the region of amplification on chromosome 8 shown in (b). The chromosome 9 signals appear to flank the chromosome 8 material on this chromosome. Amplification of the region around DHFR* is indicated by the large hybridization signal from CTD-3145B15. The amplified DNA was determined to be located on chromosome 9 by hybridization of RP11-62H18 to 9pter (not shown). Thus, material from 9qter appears to be amplified *in situ *on chromosome 9 and additional copies of material from the chromosome 9 amplicon are present on a separate chromosome together with amplified DNA from chromosome 8. **(d, e) **Copy number profiles of chromosomes 19 (d) and 8 (e). **(f) **Organization of the amplicon. RP11-691H23 (red) maps near the DHFR* integration site on chromosome 19 and RP11-175H20 (green) is one of the clones from the amplicon on chromosome 8 shown in (e). The chromosome 19 signals appear to flank a number of copies of chromosome 8, which could be as many as eight copies, since the CGH log_2 _ratio = ~2. Two additional copies of RP11-691H23, mapping near DHFR* on chromosome 19, were also present on chromosome 19 (data not shown). Thus, amplified DNA near the DHFR* integration site is present and independently amplified on two chromosomes. **(g, h) **1M-42_9 CGH profile (chromosome 8) showing the DHFR* amplicon and its organization as determined by FISH. BAC clone RP11-91O11 (red) maps near the DHFR* integration site and is co-amplified with the distal part of chromosome 8 (RP11-237F24, green). The chromosomal region between the two co-amplified regions was lost. **(i, j) **1M-42_2 CGH profile (chromosome 8) and FISH analysis showing that the two regions of chromosome 8 were amplified as two independent amplicons on different chromosomes.

Amplified DNA can be present in various forms, including double minutes, amplified regions on a chromosome, which may be cytogenetically visible as a homogeneously staining region (HSR), or distributed across the genome [19]. The organization of 13 amplicons from five integration sites was investigated using FISH. All of the DHFR* amplicons in methotrexate resistant cells were present as amplified DNA on one or two chromosomes. In only one case (1M-89_6) was the amplified DNA present as double minutes in some cells rather than integrated into a chromosome. Hybridization of differentially labeled FISH probes from the amplicons revealed that eight of the chromosomal amplicons were organized as repeated units in inverted orientation (Figures 2 and 3). The organization of the remaining four was not determined. In five cases there were also copy number losses distal to DHFR* in the copy number profile. These observations are consistent with amplification being initiated by a double strand break distal to DHFR*, followed by breakage-fusion-bridge cycles. In two independent methotrexate resistant clones from 1M-57 in which DHFR* is integrated near the chromosome 3q telomere, we observed amplicons containing both the DHFR* integration site on 3q as well as amplification of 3pter. Cytogenetic analysis revealed the presence of ring chromosomes and patterns of hybridization of FISH probes on linear and ring chromosomes consistent with amplification occurring by a breakage-fusion-bridge process involving fusion of 3pter and 3qter (Figure 2).

Translocation prior to amplification also appears to have occurred in four resistant colonies in which the amplicons were formed from two separate genomic regions that were co-amplified in inverted repeats. In the two examples shown in Figures 3a–f, hybridization of FISH probes is consistent with different regions of chromosome 8 being translocated onto the chromosome carrying DHFR* followed by co-amplification. In both cases distal parts of chromosome 8 were lost. Amplicons containing two or more separate regions of the same chromosome organized as inverted repeats were also observed (Figure 3g,h). On the other hand, the contiguous genomic region of amplified DNA on 8q in 1M-42_2 was present on two different chromosomes (Figure 3i,j). In these cells ring chromosomes were also present.

### Fragile sites and the propensity to amplify

**Figure 4:**
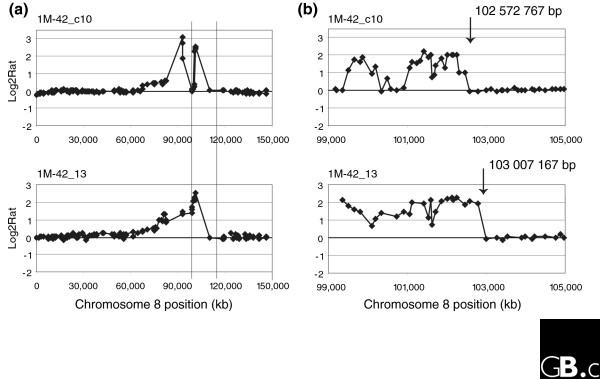
Recurrent amplicon boundaries in 1M-42 methotrexate resistant clones and fragile sites. **(a) **Chromosome 8 copy number profiles at approximately 1.4 Mb resolution. The vertical lines indicate the region from 99 to 105 Mb on chromosome 8 shown for **(b) **hybridization of these same DNAs to the 32K genome tiling array.

The integration sites varied in the frequency with which resistant clones amplified DHFR*. The 1M-42 integration site was unique in that DHFR* was amplified in almost all resistant clones (13/15), which was significantly more frequent than any other site (test for homogeneity of binomial proportion, *p *= 0.0002). Although the clones varied with respect to the regions of chromosome 8 that were amplified together with DHFR*, they all shared similar distal amplicon boundaries mapping between RP11-10G10 and CTD-2013D21. Higher resolution mapping on the 32K bacterial artificial chromosome (BAC) genome tiling path array [20] allowed the boundaries of five amplicons to be mapped more precisely (Figure 4). Four of the boundaries were positioned in a 1 Mb region between RP11-375I14 and RP11-97D1 (102322735 to 103386096 base-pairs (bp), May 2004 freeze, Additional data file 3).

The consistent and recurrent location of amplicon boundaries to a limited region prompted us to investigate the possible involvement of fragile sites in initiating amplification. The integration site of DHFR* at 102,162,871 bp on chromosome 8 is close to several fragile sites, including the aphidicolin sensitive sites FRA8B, FRA8C and FRA8D and the distamycin A inducible site, FRA8E. A rare folate sensitive site, FRA8A, has also been localized to 8q22.3 (101,600-106,200 kb). As cells are being deprived of folates by challenge with methotrexate, a potential role for the folate sensitive fragile site, FRA8A seemed possible. Therefore, we sought evidence of a methotrexate induced fragile site in the region of the recurrent boundary of the 1M-42 amplicons in HCT116+chr3 cells. Metaphase spreads prepared from cells exposed to methotrexate for 24 hours were hybridized with FISH probes labeled with Cy3 (RP11-10G10) and fluoro-isothiocyanate (FITC; CTD-2013D21). Although rare metaphases were observed in which hybridization signals from these BACs appeared to bracket a fragile site, these experiments were inconclusive due to the very low frequency with which such patterns were seen.

Seven of the 1M-42 amplicons contained more than one peak, indicating that breakage does not occur exclusively at one site on chromosome 8. Another frequent site of copy number transition occurred in the 5.8 Mb region between RP11-27I15 and RP11-238H10, which is included within the cytogenetically assigned position of the aphidicolin sensitive site FRA8B at 8q22.1. Nevertheless, we were unable to obtain evidence that induction of aphidicolin fragile sites near DHFR* in 1M-42 cells promotes amplification, since exposure of 1M-42 cells to aphidicolin for 24 hours prior to methotrexate challenge did not result in a statistically significant difference in the number of resistant clones compared to cells without aphidicolin pretreatment.

### Amplification and gene expression

**Figure 5:**
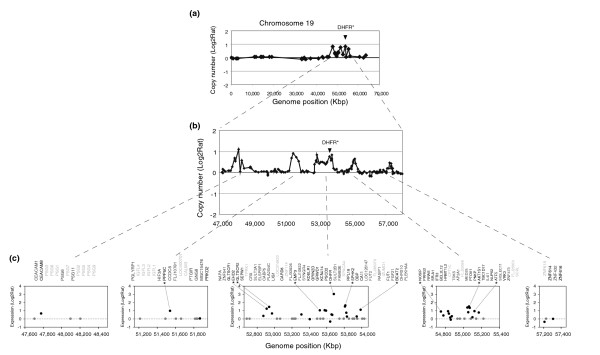
Expression of genes mapping to the amplicon from methotrexate resistant colony 1M-89_6. **(a) **Chromosome 19 copy number profile at approximately 1.4 Mb resolution (HumArray3.0) shows four discrete regions of amplification. **(b) **Chromosome 19 copy number profile from the 32K BAC genome tiling path array in the amplified region showing the four regions of amplification detected on the lower resolution array and an additional small region distal to the others. The regions, ranging in size from 0.2 to 1.2 Mb, were amplified together in some cells as double minutes, while in others the amplified DNA was integrated into different chromosomes and present as a homogeneously staining region. Both copies of chromosome 19 were retained without rearrangement. As all regions are included together on the double minutes, their formation may have occurred by joining of broken pieces of DNA subsequent to resolution of stalled replication forks [63]. **(c) **Expression levels of genes mapping to the five amplified regions plotted according to position on the human genome sequence. Expression levels are shown as the log_2 _ratios of the signal intensities after hybridization of Cy3 labeled cDNA from the methotrexate resistant colony and Cy5 labeled cDNA from the untreated parent 1M-89 as reference. Shown is the list of genes in genomic order that map to the amplified regions according to the RefSeq database [64]. Genes that are expressed in this cell line with or without exposure to methotrexate are labeled in black and expression levels denoted by black dots. Genes present on the array, but not expressed in untreated or resistant cells, are colored by dark gray and represented by dots of the same color with zero change in expression. Genes not present on the array are listed in the upper line in light gray. Genes with log_2 _fold change >0.8 are indicated with an asterisk.

A primary reason for amplification of a gene under permanent selection pressure is its increased expression resulting from the copy number increase. A question frequently asked about amplified genes is whether they are the driver gene for amplification or are they simply passengers. In our model system, the presence of DHFR* significantly increases the IC-50 for cells challenged with methotrexate, suggesting that it is the driver gene for its amplicons. Moreover, expression of DHFR* as measured by quantitative RT-PCR is positively correlated with copy number determined by array CGH (*p *< 0.05), providing further support for DHFR* as the driver gene for amplification, regardless of site of integration. On the other hand, the amplicons always span regions much larger than DHFR* and include other genes (Figure 5); therefore, we asked which neighboring genes in the amplicons are also up-regulated by copy number. Twelve methotrexate resistant colonies (four different integration sites) were selected for microarray analysis of gene expression at the mRNA level (Additional data file 4). All of the resistant clones contained amplification of the region containing DHFR* and some also co-amplified additional regions. Considering only genes located within the 12 regions of amplification and with measured expression levels in both amplified and non-amplified samples (*n *= 370; Additional data file 5), we found that the mean expression levels of 139 were up-regulated when amplified compared to mean expression levels in samples without amplification (log_2 _fold change > 0.8), with the likelihood of up-regulation appearing to be independent of proximity to DHFR*. An additional 13 genes were highly expressed in methotrexate resistant samples without amplification (log_2 _ratio > 0.8) and 9/13 also showed additional modest increases in expression in samples with amplification. Although these genes could contribute to methotrexate resistance when amplified, we were unable to demonstrate any increase in IC-50 for methotrexate or growth advantage when two randomly selected amplified genes with positive correlation of expression with copy number (*POLR2K *and LOC157567) were overexpressed in HCT116+chr3 cells. Similarly, overexpression of *MYC*, which was co-amplified with DHFR* in a number of resistant cells from different integration sites, did not significantly alter the IC-50 for methotrexate or provide a proliferative advantage (data not shown). Thus, DHFR* appears to be the major driver gene for amplification, although we cannot rule out that one or more of the genes included in the amplicon could also provide a significant contribution to methotrexate resistance.

### Response to methotrexate is independent of site of integration and amplification

To investigate further the question of the contribution of co-amplified genes to the overall response of cells to methotrexate, we asked whether expression profiles of methotrexate resistant colonies varied with respect to integration site. Unsupervised hierarchical clustering of the 12 samples with amplification revealed two major clusters, indicating at least two responses to methotrexate. Samples did not separate according to integration site, however, further suggesting that co-amplified genes do not contribute significantly to the overall response to methotrexate.

**Figure 6:**
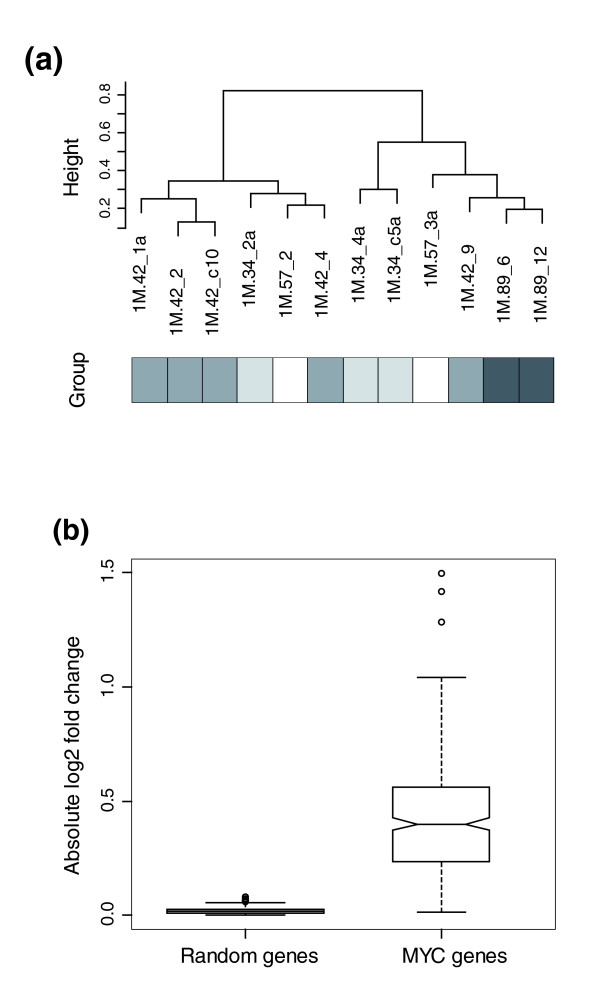
Expression profiling of 12 methotrexate resistant colonies with amplification of DHFR*. **(a) **Unsupervised hierarchical clustering (Pearson) of genes with variable expression across the data set (SD ≥ 0.3) and present in >75% of samples (3,931 genes). Methotrexate resistant colonies represent four different integration sites, indicated by the shaded boxes below the dendrogram. **(b) **Comparison of expression of *MYC *target genes in the two clusters. The median absolute log_2 _ratio change in expression of *MYC *target genes in the right cluster compared to the left cluster is significantly higher than a similar comparison using 1,000 sets of 380 randomly selected genes (*p *= 2.2e-16).

One notable difference between clusters was the presence of two samples with focal amplification of *MYC *in the right cluster (1M-34_c5 and 1M-42_9; Figure 6a,b). In general, samples in the right cluster with or without amplification of *MYC *showed higher *MYC *expression than those in the left cluster (log_2 _ratio change between clusters = 1.42). Using the *MYC *target gene database [21], we identified 380 human *MYC *target genes among the 3,931 variably expressed genes used for clustering. The median absolute log_2 _ratio change in expression of these target genes in the right cluster compared to the left cluster was significantly higher than a similar comparison using 1,000 sets of 380 randomly selected genes (*p *= 2.2e-16). Thus, differential expression of *MYC *and *MYC *target genes is one of the distinguishing features of the overall response of cells with amplified DHFR* to challenge with methotrexate, irrespective of integration site.

## Discussion

In order to investigate the effect of genome position on the propensity to amplify, we integrated a single copy of a mutant form of *DHFR *fused to the gene encoding EGFP (DHFR*) into different positions in the genome of HCT116+chr3 cells by retroviral transfer, challenged cells with methotrexate and then studied the genomic alterations arising in drug resistant cells. Since DHFR* confers greater resistance to methotrexate than the endogenous wild-type *DHFR*, we expected that increased copy number of this gene would be found in the methotrexate resistant cells, rather than the endogenous gene. This expectation was met, as the majority of the resistant colonies contained either gains or amplifications of the locus. Furthermore, DHFR* appeared to be the driver gene for the copy number change, since DHFR* mRNA levels were positively correlated with DHFR* copy number. Nevertheless, in this simple model system, we observed that at four different DHFR* insertion sites, approximately one-third of neighboring genes were also up-regulated when amplified along with DHFR*. It is unlikely that so many of these genes mapping to four different random locations would also be driver genes for amplification. Moreover, expression profiling divided methotrexate resistant cells into two groups independent of integration site, suggesting that neighboring genes in the amplicons, even though up-regulated by copy number increases, did not play a major role in the drug resistant phenotype. Taken together, these observations suggest that about one-third of genes can be regulated by copy number, which is consistent with global expression array profiling of tumors. If these four regions are representative of the genome as a whole, then a significant proportion of the amplified genes in tumors are also likely to be passengers. These observations have implications for studies of amplicons in tumors. Passenger genes will confound efforts to identify candidate oncogenes by expression analyses alone. For example, several candidate driver genes for amplification are thought to be present in amplicons in human cancers, including 8p11-p12 and 17q12 in breast cancer [22-24], 7p11.2 in glioblastoma [25] or 6p22 in bladder cancer [26], because gene expression is correlated with copy number. Further functional studies will be necessary to determine which overexpressed genes in the amplicons contribute to tumor development. On the other hand, *BIRC2 *and *YAP1*, two genes present in a narrow amplicon in oral squamous cell carcinomas [27], esophageal squamous cell carcinoma [28], and lung [29], pancreatic [30], and hepatocellular carcinomas, have recently been shown to collaboratively promote tumor formation in mice [31], indicating that the extent of some tumor amplicons may be determined by selection for multiple neighboring collaborating oncogenes.

A distinguishing feature of the expression profiles of methotrexate resistant cells was the expression of *MYC *target genes. Co-amplification of *MYC *irrespective of integration site also suggests that it played a role in the response to methotrexate; however, the exact mechanism whereby *MYC *contributes to resistance is not known. It does not appear that overexpression of *MYC *contributes to resistance simply by promoting progression through the cell cycle or genome instability, since overexpression of *MYC *prior to methotrexate challenge did not enhance drug resistance or proliferation. On the other hand, up-regulation of *MYC *expression may contribute to drug resistance by enhancing the capability of cells to evade checkpoints. For example, overexpression of *MYC *abrogates a p53-dependent cell cycle arrest in REF52 cells exposed to *N*-(phosphonacetyl)-L-aspartate (PALA), an inhibitor of pyrimidine nucleotide synthesis, allowing resistant cells to arise from these normally non-permissive cells [5].

The numbers or types of genomic alterations in resistant cells varied among the integration sites; however, they were not related to either expression levels or sensitivity to methotrexate (Table 1). Previous studies in hamster cells, yeast and protozoa have highlighted the association of genome position with the frequency of methotrexate resistant cells and repeated sequences have been implicated in promoting amplification in response to methotrexate challenge in yeast and *Leishmania *[10,32,33]. Moreover, in *Leishmania *the response to methotrexate differs among species and it was possible to show that not only was position important, but also the nature of the gene under selection; that is, amplification of the gene conferring the higher level of resistance was observed more frequently in resistant cells regardless of position [34] as observed here in human cells for DHFR*.

In spite of the fact that DHFR* confers greater resistance to methotrexate, DHFR* was not altered in copy number in resistant colonies from two integration sites, while no resistant colonies were recovered from a third integration site. Expression levels of DHFR* in these integrants, measured indirectly as EGFP expression, were in the middle of the range of expression levels for all integration sites as was sensitivity measured as IC-50. Furthermore, no variations in DHFR* were detected by sequencing DNA amplified from genomic DNA of the three clones. Thus, it appears that failure to observe copy number changes of DHFR* in these integration sites is related to genome position. We hypothesize that these regions harbor genes that, if amplified, would cause cell cycle arrest or cell death. The 1M-73 integration site is within <700 bp of *CDKN1B *(p27Kip1), a negative regulator of the cell cycle [35]. Overexpression of *CDKN1B *due to copy number increase of the locus could suppress growth and abrogate any advantage that might have been conferred by increased copy number and expression of DHFR*. Similarly, in 1M-84, DHFR* is integrated between *PCGF2 *(Mel-18) and *PSMB3*, approximately 1 Mb proximal to *ERBB2*, a gene frequently amplified in cancer. Nevertheless, *PCGF2 *and *PSMB3 *are rarely amplified with *ERBB2 *[36] in tumors, and *PCGF2 *has been reported to be a tumor suppressor [37,38]. Moreover, a recent report indicates that overexpression of *PCGF2 *leads to down regulation of *MYC *and senescence in human fibroblasts [39]. Thus, it is likely that amplification of DHFR* at the 1M-84 insertion site would be deleterious to cells exposed to methotrexate due to proximity to, and thus co-amplification with, *PCGF2*. These considerations suggest that there may be selection pressure from neighboring growth inhibitory genes on the positions of amplicon boundaries.

**Figure 7:**
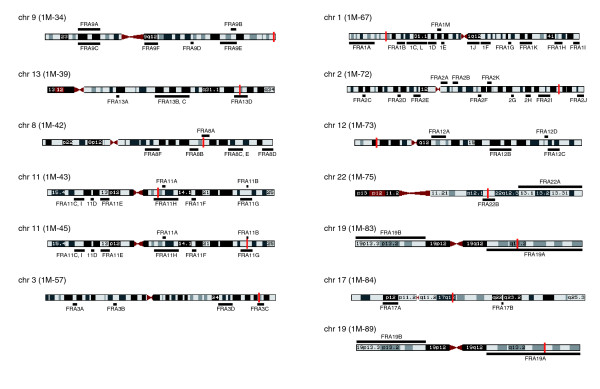
Positions of DHFR* integration sites and fragile sites. The DHFR* integration sites were mapped onto the genome using BLAT [46] and are shown as a red bar on the chromosome ideogram from the UCSC genome browser [45]. Common fragile sites are shown under the chromosome and are mapped according to positions reported in NCBI Entrez and the literature [65-77]. Rare folate sensitive fragile sites are shown above the chromosome. Note that the 1M-45DHFR* integration site mapped just proximal to FRA11B, another folate sensitive fragile site; however, no copy number transitions or amplicon boundaries mapped to this region. Since FRA11B is a RFS, it may not be expressed in HCT116.

Copy number changes involving DHFR* encompassed regions much larger than the integrated DNA and, in most cases, the boundaries of the copy number changes were not recurrent as we had found previously for the endogenous locus on 5q13 [17]. At the 1M-42 integration site, however, we did observe a high frequency of amplification and recurrent copy number transitions or amplicon boundaries at RP11-238H10 and CTD-2013D21, which occurred in 50-70% of resistant colonies. A role for expression of fragile sites in promoting amplification and setting boundaries to the amplicons in tumor genomes was suggested more than 20 years ago [40,41]. Although several fragile sites, including the folate sensitive fragile site FRA8A, map near the 1M-42 integration site on 8q22 (Figure 7), we did not find that FRA8A was expressed at sufficiently high frequency in HCT116 cells for it to be mapped by FISH. Nevertheless, even low frequency expression of fragile sites in cells exposed to methotrexate leading to an increased rate of breakage in close proximity to a DHFR* integration site could be sufficient to enhance the probability of amplification and selection for cells with recurrent amplicon boundaries. Thus, in the 1M-42 resistant colonies, induction of FRA8A by exposure to methotrexate could have provided a double-strand DNA break in close proximity to DHFR*, which initiated amplification of this locus and subsequent survival advantage of these cells in the presence of methotrexate. Similarly, the association of folate deficiency with breast cancer suggests that in individuals who express folate sensitive fragile sites, breakage may occur at higher frequency at these loci, leading to chromosomal rearrangements promoting tumor development. We have, however, been unable to find evidence that fragile sites play a more general role in setting aberration boundaries in breast cancer (that is, using 1.5 Mb resolution array CGH data and looking over 5 Mb windows on chromosome arms, we found no evidence that fragile sites are associated with amplicon boundaries in breast tumors). We note that these analyses are currently hampered by the relatively low resolution mapping of both copy number aberration breakpoints and fragile sites. On the other hand, it is noteworthy that amplification was significantly more frequent at the 1M-42 integration site, suggesting that, if not fragile sites, then some, as yet undefined genomic features may have influenced the propensity to amplify. Similar genomic characteristics may also play a role in setting boundaries for amplicons in human tumors.

## Conclusion

Altered expression of genes can occur by a number of mechanisms, of which copy number change is only one. In the simple system we have used to study genome position effects, cells are being challenged by a single agent and also offered a gene that can provide resistance, unlike the development of human tumors in which, as normal cells evolve to cancer cells, many barriers must be breached. There are many ways to alter signaling or checkpoint pathways, resulting in evasion of apoptosis and enhanced proliferation. Increasing expression of appropriate genes in these pathways by copy number changes is only one mechanism. The observations reported here on a model system translated to tumor development suggest that the nature of the gene under selection for growth advantage and genome context are, together, factors influencing the propensity to amplify. In addition, it is likely that the nature of the challenges experienced by the nascent cancer cell (for example, folate deprivation), individual variation (for example, frequency of expression of various fragile sites), and the types of genomic instability that may be present also influence which genes will be altered in expression by copy number changes.

## Materials and methods

### Cloning, cell culture, infection

The human colorectal HCT116+chr3 cell line (gift from Dr CR Boland) was maintained in DME H-21 media supplemented with 10% fetal bovine serum, 1 × non-essential amino acids, 100 U/ml penicillin and 100 μg/ml Streptomycin, and 400 μg/ml G418 (Invitrogen, Carlsbad, CA, USA). The MMLV vector containing the double mutant dihydrofolate reductase gene (L22F, F31S) fused to the gene encoding EGFP was a generous gift from Dr J Bertino, Cancer Institute, New Jersey. Using this vector, we prepared a *DHFR *variant with a single mutation (L22F) fused to the *EGFP *gene, which we refer to as DHFR*.

HCT116+chr3 genomic DNA was used as a template for amplifying the *DHFR *promoter and 5' segment of the gene (from -461 to +23 relative to the ATG translation initiation codon [42]) with primers containing *Esp*3I restriction sites at the 5' end. The 3'sequence of DHFR* (from +20 relative to the ATG translation initiation codon) was PCR amplified from the vector (primers also contained *Esp*3I restriction sites at the 5' end). Both PCR products were cleaved with *Esp*3I restriction enzyme, ligated together and cloned into pLPCX retroviral vector (Clontech, Mountain View, CA, USA), which had been linearized by digestion with *Nco *and *Hin*dIII restriction enzymes, resulting in removal of the CMV promoter from the vector.

To deliver DHFR* into HCT116+chr3 cells, we used the Phoenix retroviral system (Orbigen, San Diego, CA, USA). HCT116+chr3 cells were sparsely seeded into 6-well plates (10^3 ^cells per well) and infected with diluted viral particles the following day (MOI < 0.1). Two days after infection, resistant colonies were selected with puromycin (0.3 μg/ml media) for seven days. Individual colonies were expanded as separate integration site clones (1M-XX) without puromycin.

To express *POLR2K *and LOC157567 in HCT116+chr3, cDNA clones (MSH1010-74358 and MHS1010-9205152, respectively) were purchased from Open Biosystems (Huntsville, AL, USA). The coding sequence was amplified with primers containing *Esp*3I restriction sites and cloned into the *Hin*dIII site of the pLHCX vector (Clontech). To overexpress *MYC*, the cDNA was cloned into the pBABE-hygro vector. The integrity of the constructs was verified by sequencing and the Phoenix retroviral system was used to deliver the genes into HCT116+chr3 cells. Two days after infection, cells were exposed to hygromycin (400 ng/ml media) for 7 days to select pools of cells with stable insertion.

### Methotrexate sensitivity

Each clone was seeded into one 96-well plate (400 cells per well) leaving outer wells without cells, only with media. The next day different concentrations of methotrexate were added and cells were cultured for 72 hours. After three days, half of the media was replaced with fresh media with the same methotrexate concentration and cells cultured for another three days. Cells were washing gently with 1 × phosphate-buffered saline and plates were stored at 80°C prior to measuring cell proliferation using the CyQUANT Cell Proliferation Assay (Invitrogen, Carlsbad, CA, USA). The inhibition concentration (IC-50) was calculated for each integration site clone.

### Selection of methotrexate resistant colonies

Individual clones were seeded into 6-well plates (10^3 ^cells per well) and grown until the population reached approximately 5 × 10^5 ^cells in each well. Then the cells from each well were counted and seeded into 100 mm dishes (4 × 10^5 ^cells per dish). The next day different concentrations of methotrexate (3-4 × IC-50, depending on clone sensitivity to methotrexate) were added to the cells. The medium was replaced every third day and after four weeks one colony from each dish was picked into a 12-well plate and then expanded into T75 culture flasks. DNA was isolated from a quarter of the cells from each T75 flask, and another quarter of the cells was expanded for isolation of RNA, proteins and metaphase spreads. The remaining half of the cells was frozen in liquid nitrogen. In other experiments, colonies were selected as above and then fixed and stained with crystal violet after four weeks to determine the number of resistant colonies.

### Induction of fragile sites

Individual clones were seeded into 6-well plates (10^3 ^cells per well) and grown until the population reached approximately 10^6 ^cells in each well. Then the cells from each well were counted and seeded into two 100 mm dishes (4 × 10^5 ^cells per dish). After six hours, fragile site expression was induced in one of the two dishes by adding aphidicolin (at a final concentration of 0.15 μM). After 24 hours, aphidicolin was removed and methotrexate was added to all dishes (same concentration as used for selection of methotrexate resistant colonies). Colonies were fixed and stained with crystal violet after four weeks. Folate sensitive fragile sites were induced by adding methotrexate to HCT116+chr3 cells (25 nM) or the 1M-42 clone (75 nM). After 24 hours, metaphase spreads were prepared according to standard protocols.

### Inverse PCR

Inverse PCR was performed as described previously [43,44] with slight modification as described in Additional data file 1. To map the integration sites on the genome sequence, the PCR products amplified in a second nested PCR were sequenced and mapped onto the human genome sequence using BLAT (UCSC Genome Browser, May 2004 freeze [45,46]). Methodological details and sequences obtained by inversion PCR for each insertion site are provided in Additional data file 1.

### Array CGH

Array CGH was carried out as described previously [17]. Briefly, genomic DNA was isolated from one quarter of the cells from a T75 flask by incubation overnight at 55°C in 3 ml of 1 × TE buffer (pH 7.5) supplemented with 0.5 % SDS and 0.1 μg/μl proteinase K (Roche Diagnostics Corporation, Roche Applied Science, Indianapolis, IN, USA), followed by ethanol precipitation after 24 hours. The DNA was dissolved in 100 μl of H_2_O. Genomic DNA (600 ng) was labeled by random priming to incorporate Cy3 or Cy5 dCTP in a 50 μl reaction and the labeled test and reference DNAs together with 100 μg human Cot-1 DNA were hybridized for approximately 48 hours at 37°C to arrays of 2,464 BAC clones, each printed in triplicate (HumArray2.0, 3.1, UCSF Comprehensive Cancer Center Microarray Core). Whole genome tiling path arrays containing 32,145 BAC clones [20] from the UCSF Comprehensive Cancer Center Microarray Core were used for more detailed analysis of some amplicons. A custom built CCD camera system was used to acquire 16 bit 1,024 × 1,024 pixel DAPI, Cy3 and Cy5 images [47,48]. Array data are available in Additional data files 2 and 3 and at NCBI Gene Expression Omnibus [49]. GSE6262 contains 95 CGH profiles of methotrexate resistant colonies and also untreated controls (HumArray2.0, 3.1). GSE6360 contains six CGH profiles (32K BAC tiling path array).

### Expression microarrays

Test (20 μg) and reference (20 μg) RNA were reverse transcribed in the presence of aminoallyl-dUTP followed by coupling with Cy3 or Cy5 dyes. The untreated integration site clone was used as the reference (Cy5 labeled cDNA) for each of the hybridizations with its respective resistant colonies (Cy3 labeled cDNA). Hybridizations were carried out on arrays of printed long oligonucleotides (70 mers) containing 21,000 elements (Operon V2.0 [50]). Array images were analyzed using UCSF SPOT software [51]. Expression data were filtered to exclude all spots with foreground intensity below 500 units in the Cy3 or Cy5 channel and were subsequently median normalized and log_2 _transformed. Data are available in Additional data file 4 and at NCBI GEO (GSE6262).

### Copy number analysis

Image and data analyses were carried out using UCSF SPOT [51] and SPROC software, as described previously [17]. We also made corrections for two well recognized sources of variation in ratios; the observed dependence on location of clones on the array (spatial or geometric variation) [52], and the dependence of ratios on GC content [53]. To correct for geometrical dependence of the log_2 _ratios over the array, we took advantage of the fact that neighboring loci in the genome are, for the most part, likely to have the same copy number [54]. We used backfitting [55] to iteratively obtain an estimate of the systematic spatial variation of the log_2 _ratios over the array, which was then subtracted from the original log_2 _ratios (Additional data file 6). The log_2 _ratios were corrected for GC content using a loess method [56]. That these procedures result in a reduction in the apparent 'noise' in the ratio profiles, but do not alter the ratios, is clearly seen by a side-by-side comparison with the array data prior to correction (Figures B and C in Additional data file 6).

### Elucidation of copy number changes specific to methotrexate resistant cells

Although the copy number differences between a methotrexate resistant and untreated HCT116 cell line are easily distinguished by visible inspection of the copy number profiles, we developed an objective method to identify the copy number changes acquired by the drug resistant cells. Since the HCT116 cells have a small number of low level copy number changes (Figure 1a), it is necessary to exclude them from the analysis by 'subtracting out' aberrations that were present in the cell population prior to exposure to methotrexate. Therefore, to identify the copy number changes specific to methotrexate resistant cells, aberrations in the copy number profile of the untreated cells for each DHFR* insertion site (called 'parent') were subtracted from the resistant cell profiles for each colony from that insertion site (called 'colony'). However, since the amplitudes of the ratio changes in array CGH may vary among different measurements of related samples due, for example, to incomplete suppression of repeated sequences (Figure 1 in Additional data file 7), we implemented a correction procedure that took into account the differences in amplitude between the array CGH profile of a resistant colony relative to the parent. The correction was based on the assumption that when biases equally affect test and reference signals, a copy number change and the observed copy number linear ratio are linearly related, but the slope may be reduced as the bias increases [57]. Taking into account the known homozygous deletion on chromosome 16p in HCT116, a robust linear model was fit to the linear ratio of the colony and the parent with the colony as the response (Figure 2a in Additional data file 7). A weight of 500 was given to the homozygous deletion (using a cutoff of linear ratio <0.25). The large weight was necessary because of the small number of arrayed BACs reporting homozygous deletion (mean four BACs per sample) compared to the total number of arrayed BACs used for the analysis (2,056-2,282, before parent subtraction). The fitted value gave the parental ratio after correcting for bias. Thus, the ratio of the colony to the fitted parent ratio was considered the colony specific copy number change (Figure 2b in Additional data file 7). A comparison of this procedure with and without using the spatial correction procedure described in the previous section is shown in the upper panels in part (b) of Additional data file 6. Since we considered each insertion site separately and required that ratios on arrayed BACs be present in both the parent and the colony for each insertion site, the number of BACs remaining after this analysis varied among the different insertion site parents and their colonies (1705-2099).

After subtraction of the linear parent ratios, the array CGH data were converted to log_2 _and analyzed as reported previously [58] using circular binary segmentation (CBS) [59] with default parameters to translate experimental intensity measurements into regions of equal copy number as implemented in the DNAcopy R/Bioconductor package [60]. Missing values for BACs mapping within segmented regions of equal copy number were imputed by using the value of the corresponding segment. A few clones with missing values were located between segmented regions and their values were imputed using the maximum value of the two flanking segments. Thus, each array clone was assigned a segment value referred to as its 'smoothed' value. The scaled median absolute deviation (MAD) of the difference between the observed and smoothed values was used to estimate the sample-specific experimental variation. All the samples had MAD less than 0.15. Due to additional random noise introduced when subtracting the parent from the resistant colonies, the smoothed values from CBS were further 'merged' using the MergeLevels procedure [61]. In this process, segmental values across the genome were merged to create a common set of copy number levels for each sample. The minimum and maximum criteria used in MergeLevels were 0.05 and 0.5, respectively.

To identify single technical or biological outliers, such as high level amplifications, the presence of the outliers within a segment was allowed by assigning the original observed log_2 _ratio to the clones for which the observed values were more than four tumor-specific MAD away from the smoothed values. The amplification status for a clone was then determined as described previously [58] by considering the width of the segment to which that clone belonged (0, if an outlier) and a minimum difference between the smoothed value of the clone (observed value, if an outlier) and the segment means of the neighboring segments. The clone was declared amplified if it belonged to the segment spanning less than 20 Mb and the minimum difference was greater than exp(-x3) where x is the final merged value for the clone.

#### Expression of genes in amplicons

For 12 samples the borders of the regions determined to have been amplified by array CGH were further refined by manually incorporating additional data from the whole genome tiling path arrays and FISH experiments. The border of each amplicon was set between the last not amplified BAC clone and the first amplified BAC clone. Expression probes were first filtered for ones with missing annotation, resulting in 19,777 probes, which were then mapped to the genome sequence. From these mapped probes, a list of expression probes included in all the amplicons within the samples was compiled manually, resulting in 685 expression probes, of which 370 provided measured expression in both amplified and non-amplified samples. To assess whether a gene was up-regulated when amplified, the mean expression levels were calculated in samples with and without amplification of the gene. Mean expression levels with and without amplification were compared and those with log_2 _fold change >0.8 were considered to be up-regulated when amplified.

#### Analysis of clustering of gene expression driven by *MYC *target genes

Genes with variable expression across the data set (standard deviation (SD) ≥ 0.3) and present in >75% of samples were identified and filtered for those with missing annotation. The samples were clustered based on the remaining clones (3,931) using the Pearson correlation distance metric and complete linkage. Human '*MYC *target' genes (1,287) were identified by reference to the *MYC *database [21], and 380 were included in the set of 3,931 variably expressed genes. The fold change in expression of 380 *MYC *genes between the two sample clusters was computed and compared to the median log_2 _fold change for 1,000 selections of 380 random genes.

### Quantitative RT-PCR

Real-time quantitative RT-PCR was performed on RNA isolated from methotrexate resistant colonies and untreated insertion site clones using Trizol reagent (Invitrogen) and treatment with DNase (Promega, Madison, WI, USA) as previously described [62] in the UCSF Comprehensive Cancer Center Genome Analysis Shared Resource Facility. Primers (forward, 5'CGTAAACGGCCACAAGTTCAG, reverse, 5'GGGTCAGCTTGCCGTAGGT) and probe (5'6FAM-CGCCCTCGCCCTCGC-BHQ_1) for EGFP were designed using GenScript Primer Design (GenScript Corp., Piscataway, NJ, USA). The ABI Assays-on-Demand were used to confirm overexpression of *MYC*, *POLR2K *and LOC157567 genes (ABI, Foster City, CA, USA).

### Fluorescent *in situ *hybridization

Metaphase spreads were prepared from untreated insertion site clones and methotrexate resistant colonies at the time of RNA and protein isolation. Before hybridization slides were aged in 2 × SSC at 37°C for 30 minutes, dehydrated in an ethanol series (70%, 80% and 96%) and incubated in 70% formamide, 2 × SSC at 72°C for 4 minutes to denature the DNA. Fluorescent probes were prepared by labeling 20 ng of BAC DNA by random priming to incorporate Cy3- or FITC-coupled dCTP. The labeled DNA was precipitated, pre-annealed with 30 μg Cot-1 DNA and hybridized to metaphase spreads for 48 hours at 37°C in a humid chamber. Slides were washed three times with 50% formamide, 2 × SSC at 45°C for 15 minutes, 2 × SSC for 15 minutes, 0.1 × SSC for 5 minutes, counterstained with DAPI and mounted in Vectashield (Vector Labs, Burlingame, CA, USA).

## Additional data files

The following additional data are available with the online version of this paper. Additional data file 1 shows the mapping and sequencing of DHFR* integration sites. Additional data file 2 lists array CGH log_2 _ratio data on clones for methotrexate resistant colonies and untreated DHFR* integrants. Additional data file 3 lists the Chromosome 8 array CGH dataset for five 1M-42 methotrexate resistant colonies hybridized on the 32K genome tiling path BAC array. DHFR* was integrated on chromosome 8 at 102,162,871 bp and this position is highlighted in red. All five colonies formed amplicons around the DHFR* integration site. Amplified regions are highlighted in yellow. Additional data file 4 lists Expression array data. Additional data file 5 shows the expression of genes mapping in amplicons in 12 methotrexate resistant cell lines. Additional data file 6 provides a Comparison of analysis of array data with and without spatial and GC correction. Additional data file 7 is an Example of elucidation of copy number changes specific to methotrexate resistant cells (results of the analyses for the 1M-34_c5a colony).

## Supplementary Material

Additional data file 1 Mapping and sequencing of DHFR* integration sites Click here for file

Additional data file 2 Array CGH log_2 _ratio data on clones for methotrexate resistant colonies and untreated DHFR* integrants Click here for file

Additional data file 3 DHFR* was integrated on chromosome 8 at 102,162,871 bp and this position is highlighted in red. All five colonies formed amplicons around the DHFR* integration site. Amplified regions are highlighted in yellow. Click here for file

Additional data file 4 Expression array data Click here for file

Additional data file 5 Expression of genes mapping in amplicons in 12 methotrexate resistant cell lines Click here for file

Additional data file 6 Comparison of analysis of array data with and without spatial and GC correction Click here for file

Additional data file 7 Example of elucidation of copy number changes specific to methotrexate resistant cells (results of the analyses for the 1M-34_c5a colony) Click here for file
