# Supplementary material for: Genome position and gene amplification
Source: Genome Biol. 2007 Jun 21;8(6):R120. doi: 10.1186/gb-2007-8-6-r120 (PMC2394771; doi:10.1186/gb-2007-8-6-r120)
Supplement: Additional data file 1 — Mapping and sequencing of DHFR* integration sites [file gb-2007-8-6-r120-S1.pdf]

**Mapping and Sequencing of DHFR* Insertion Sites**

**Figure 1.**

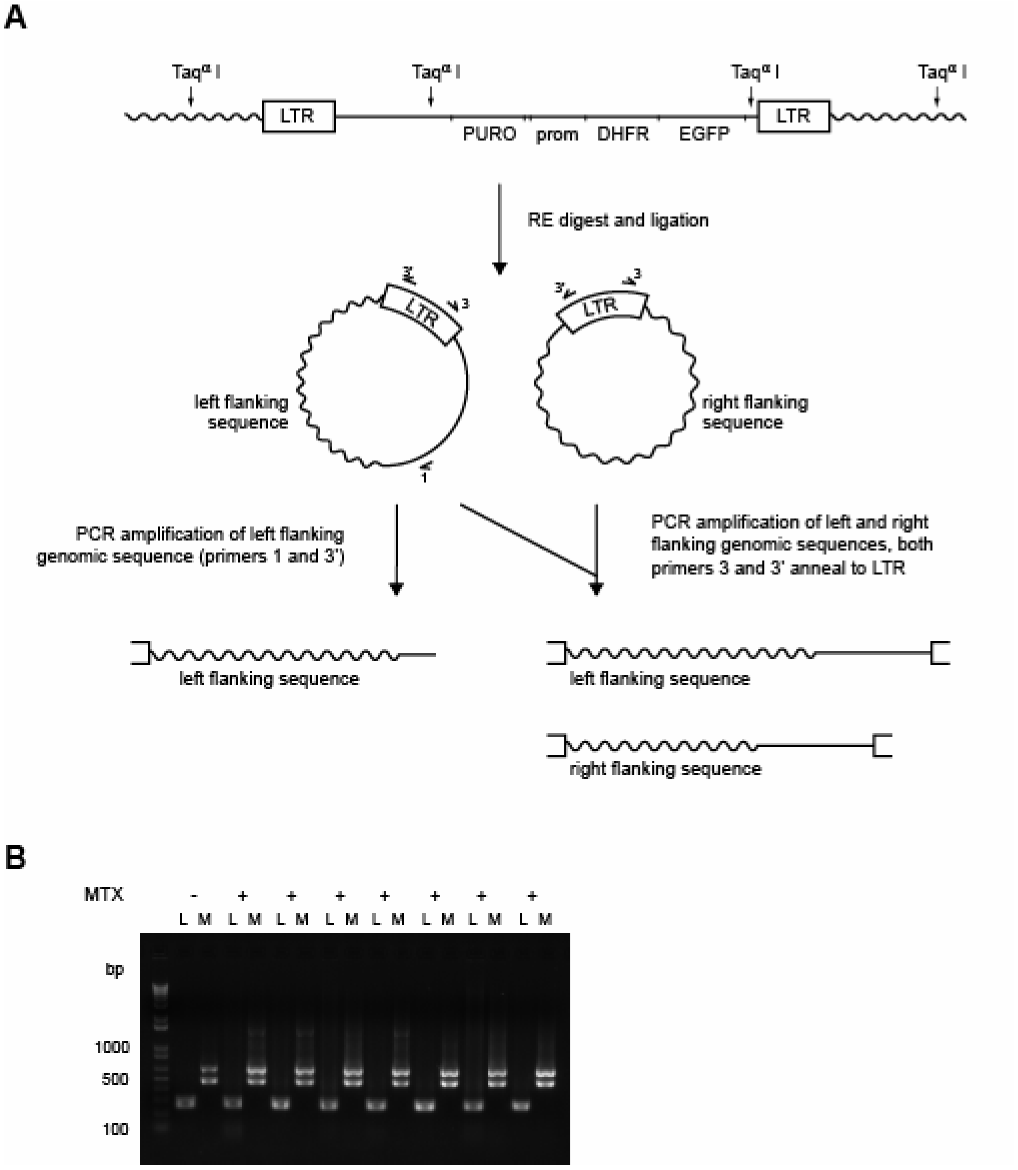


**Figure 1**. Mapping *DHFR** insertion sites by inverse PCR. (**A**) Inverse PCR schema. Inverse PCR was performed as described previously (Li et al., 1999, Kim et al., 2003) with slight modification. DNA (~3µg) was digested to completion with 60 U of $Taq_{\alpha}$ I restriction enzyme (NEB) overnight in a 40 µl reaction volume. After heat inactivation at 80º C for 20 min, the DNA was diluted to 200 µl, circularized by incubation with 1000 U of T4 DNA ligase (high concentration, NEB) at 16º C overnight. The DNA was precipitated with ethanol and re-suspended in 30 µl of water. Primary PCR was performed in two 25 µl reactions, each with 1.5 µl of the DNA template, 1x Expand Buffer 1, 350 µM of each dNTP, 10 pmol of each primer, and 1.3 U of Expand Long Template Polymerase (Roche). The primers used were Taq1, Taq3' for amplification of genomic DNA flanking the left LTR (left PCR, L) and Taq3 and Taq3' for amplification of

genomic DNA flanking both LTRs (mixed PCR, M). The plasmid sequence prohibited design of primers which would amplify only the right flanking sequence. Therefore, in the mixed PCR in addition to the band containing the right flanking genomic DNA an additional band was detected with genomic DNA flanking the left LTR (~365 bp longer PCR product than in the left PCR). The cycling conditions were 94º C for 4 min, followed by 35 cycles of 94º C for 30s, 55º C for 1 min, and 68º C for 4 min and a final extension for 10 min. The amount of primary PCR was determined semi-quantitatively by electrophoresis on a 1% agarose gel. (**B**) Left (L) and mixed (M) inverse PCR products for untreated integration site clone 1M-89 (-) and its methotrexate resistant colonies (+) following separation by gel electrophoresis. Clones were considered to have a single copy of *DHFR** integrated in the genome if they met the following criteria. First, the left inverse PCR reaction yielded a product that mapped to an exact position in the genome. Second, this integration site matched the position in the genome of all mixed inverse PCR products (see Supplementary Figure 2 for sequencing data). Third, the presence of *DHFR** was confirmed by sequencing of *DHFR** amplified by PCR from the genomic DNA isolated from individual clones (data not shown).

**Figure 2.**

1M-34 (left)

1 TTGCCAACCT ACAGGTGGGG TCTTTCACTC CTGCCTCACC CTCCCAGGTA GCTGAGATTA
61 CAGGCACCCA CCACCGCGCC TGGCTAATTT TGTACTTTT AGTAGAGACG GGATTTCACC
121 ATGTTGGCCA GGCTGGTTTT GAACTCCTGG CCTCAAGTGA TCTGCCCTCC TCAGGCTCCC
181 ACAGTGCTAG GATTGCAGGC ATGAGCCACT GTGCCCAGCT GTTGGTTGTT AATTATTTTT
241 GTTTTTTGAG ACGGAGTTTA GCTCCTGTTG CCCAGCTGGA GTGTAGTGGC ACGGTCTCGG
301 CTCACTGCAA CCTTCATCTC CCAGGTTCAC ACCATTCTCC TGCCTCAACG TCCCGAGTAG
361 CTGGGATTAC AGGCACCTGC CACCATGCCC AGCTAATTTT TTGTATTTTT AGTAGAGACA
421 GGGTTTCACC ATGTTGGCCA GGCTGGTCTC GACTCCCTTC CTCAGGTCGG GCCACAAAAA
481 CGGCCCCCAA AGTCCCTGGG ACGTCTCCCA GGGTTGCGGC CGGGTGTTCA GAACTCGTCA
541 GTTCCACCAC GGGTCCGCCA GAACGAANNN NTAGTTNAGA A

1M-39 (left)

1 GNAACCTCAG GTGGGGTCTT TCAGTTTGAT TCAAAGTCAT TGGTTCTCTT AGAGAAGGAG
61 GTCAGACCAG TTTAAGGAAC AAGGAGCAGG AGAAGAGCAA TTGAAGCCTG CACGTAGGAA
121 CTCAGATGAT AAAATCGTTT TGGAAAATAA TGAACAAGTA ACTTGAGAGC TTGTCTTGAG
181 GGTGGTATCA CAGGTTAGGC CAGGTTATTA ATTTTCCTAC TCAGCAGTAT GCCTCTTAAA
241 AAACACTTAC AGGGAAATGG ATATGTTTTT GAAAGAAATG CAATTGGCAG AAGGGAATGA
301 CGGAACTGCA TGGAGTCCAC AGGCCAGTGG ACCTGGGAGA GCAAAACTAC TTCCATGTAA
361 ATAAGAATTG CATAACTGAG AAAATACCCA AAAGATACAG CTAACAGGAC AGTGTTTAGT
421 ACACCAAAGT CGACTCCCTT CCTCAGGTCG GGCCACAAAA ACGGCCCCCA AAGTCCCTGG
481 GACGTCTCCC AGGGTTGCGG CCGGGTGTTC AGAACTCGTC AGTTCCACCA CGGGTCCGCC
541 AGATACAGAG CTAGTTAGCT AACTAGTACA GACGCAGGCC AAATNNNNAA AAAATNATGG

1M-39 (right)

1 TGTTCTTGGG AGGGTCTCCT CTGAGTGATT GACTACCCAC GCACGGGGGT CTTTCAAAAC
61 CCAACCTGAC AAGATAAATA TGAGGAAGAA AAACAAAAGA GGNCAGGGTC TGAATAATAC
121 CTTTTACNGG TNTGCANATT ATCACAGGGT TACCTATTCN NGCNCTCNGC ATATTCCAAT
181 CACCAAAAGG CNTGCNTGGA AAAANCAAAT TGGCCATGCA NTAATGCTTN CCNATTTAAA
241 GTATNATTTT GCCAATCCNG TCNCTTTTTA NCTAACNATG AAAAAAANNC AAAACACTTT
301 TTATGATTAC ANTGAANCCC TCTGNNTGTG CAGGATNANC AATNCCTTNC NNAANAANAT
361 AAAATTTTNT ATNNNCNCCN GAAAAAANGG GGCCAANAAT AACCCCCC

1M-42 (left)

1 GCAACCTACA GGTGGGGTCT TTCAGGTAGA TTGGGATGAG GATTAAAGAT AATGCATGCA
61 AAATATTTGG CACGTAGCAG GTGCTACCAA ATGCTTATTG TTGTTGTTAT TACGGAGTTC
121 AGAGCCCTTT GAGATGCTCC ACAAGTGTTT GCTGATTTGA GGGTGGCACA AAAGGCAGTT
181 TTCCCCAACA CCAATTGAAA GACTCCTTGG AGTGTGAATT TCAAGATGGT GGGGTACAGC
241 AATTTCTACC CTCTCGTCGA CTCCCTTCCT CAGGTCGGGC CACAAAAACG GCCCCCAAAG
301 TCCCTGGGAC GTCTCCCAGG GTTGCGGCCG GGTGTTCAGA ACTCGTCAGT TCCACCACGG
361 GTCCGCCAGA TACAGAGCTA GTTAGCTAAC TAGTACAGAC GCAGGCGCAA ATNCCNAAAA
421 AAATAGAA

1M-42 (right)

1 TNNCTTGGGA GGGTCTCCTC TGANTGATTG ACTACCCGTC AGCGGGGGTC TTTCATACCA
61 AGGGGGGATT ATCCGTATTT TACAGATGAG GAGACTGCAG GCCCAGCAAG GTAAAATTGT
121 TTGTCCCAGC TTTATCCTCT ATTGTATAAG TGCCAGAATT TGAATCCAGG TCTTTCTGGC
181 CCCAAGTTGC CTATGGCAAC CAAAGCCTTC CACGTCTACC TCCAGTCACT CAGTGGGTGG
241 CCTTCCTCAA ATCTTGGTTC ATCAGTCATG TCCAACACAC CGGCATGCAT CCGTGCTTAA
301 AAAGGGAAGC CAGGCAAAAT AAGCTTCCTT GGTATGCCTG TGGCCCCGTT TCCTGGTCTC
361 ATCCTGCCGT CTTTCAAGGT GAGCCTTCCA GGTAAGGGGG CTGGGCCTGG AATCCGCAAC
421 ATGCAGTCAG GAGCTGATTT TCAAAGAACC CGGTGCAGCA NGAAGACCAA GGAGACAGCC
481 GCCTCCCCTG CTCGATAAAA TAAAAGATTT TATTTAGTCT CCAGAAAAAG GGGGGAATGA
541 AAGACCCCAC CTGTAGGTTT GGCAAGCTAG CTTAANTAAC GNTTTTTTTN GNCANGGNAA
601 AGNCTAGCTT AAGTAACGCC TNTTTTNNCA AGGNA

1M-43 (left)

1 GCAACCTACA GGTGGGGTCT TTCACTCCTC CCCTTCTCCC CGCAACCCCT CTGGCCTCCA
61 ATCAGGGCCC TGCTCCGCGG CTGGCGCTCA CCCGCAACTT CAGTTCCCGT AACGGTTCCT
121 CCCGCCACCC GGCCGCTCCT GCGGATACCT CAAGCCACTA GAACTTTCTT CCACTTCCGC
181 TTCCGGGTCG CTTCAAGAGC CGCCTTTAAA AAAGAAACAT TCCACGTTGG GCACTTCTAG
241 CCAGTTAGAG CTATAGAGTT GAGGCGCCCC GGACCTCGAC TCCCTTCCTC AGGTCGGGCC
301 ACAAAAACGG CCCCCAAAGT CCCTGGGACG TCTCCCAGGG TTGCGGCCGG GTGTTCAGAA
361 CTCGTCAGTT CCACCACGGG TCCGCCAGAT ACAGAGCTAG TTAGCTAACT AGTACAGACG
421 CAGGCGCAAA

1M-45 (left)

1 GNAACCTCAG GTGGGGTCTT TCAGCCTAAG GCCCATAATT TTAGAAGAAG CAGGATTTGA
61 ATTCAGACAC CCTGGCATCT GGGTTCTGGG CCATTTCACA ATACTCCAGA TGCACCACAG
121 TATCAGTCCT CTGAACGATG ACGAAAGAAA GCATGGGTGT CCAGGCCCCA CCCTCTCACT
181 CCTCACATCC TCCCTCCATG CCTGATGTGC CCGGGGCTTG CCCAGGGAAG ATCAGAGAGA
241 GAGCACCGCT CCCCACTCCC TCAACCCAGG TACCCGCCTG GCCTGGCCTG TGGTGCCAGG
301 ACAGAGCAAA GTTCTCCAGC CTCCCCCAGC CCCGCCCAAC TCCAGTCCGT TACTTCCTCA
361 CCAATCCTTG TCCCTGCCGG TCAGAGGAGG GCACAAGGCT AGTCACCGAG ACAACAGCAC
421 AGAGCCCCTG CCAAGAGTTA CCACGGGCTC GTGAGAGGCC CCAGGTGGGG GTCAAAGGTC
481 AAGTGGGACT CACCGGTGAG GGTGGAGTCC CAGCCCTGCC CCCACCCACC TGTTCAGCCA
541 CACCTGGCCA ATGCTCCCCA TCTCTTTCAT TCCTCAGTAG AGAGCAGGAC CACTTCCTTG
601 GTGTCCCTTG GGGGGTGGCT TCAGTGGAGA GGTTCGGGGC TGANGTGAAG GGGGGAATNA
661 GAAACCCCCT CCCTCCCTGG GGATGGTCTA GGAGAAGTGC CTCCTCCCAG TCCCCAGCTT
721 CCTCCAGT

1M-45 (right)

1 NCTGTTCTTG GGAGGGTCTC CTCTGAGTGA TTGACTACCC ACGACGGGGG TCTTTCAAGG
61 CGAGTTATTT AACTTCTCGG CATGTCCCTT TTCTTAGTGG GAAAGTGAGG ATAATAAGAA
121 TACTTTCTTA CCAGATTGTT CTGAGGATTA AACTANACTA TAAGATTTTA TNTTTTCTNC
181 AGACTCCGGN GGNAGGGNGG AANGNCACAN NNAAACCTGT A

1M-57 (left)

1 GCAACCTACA GGTGGGGTCT TTCACAAGTA TTCATAAAAT CCAGGGCTCT TACTTAGTAT
61 TTCCTTAGTT CTGTGGTAGG CTGACCCTTT AATCTTGGTT GGAGTTTTCA TAATTAATGC
121 TTTGAAATAT TTTGGTGAGC TCAAGTTGTA TCTTAGGGAT CAAAGATGTG GGTCTAATCT
181 CTTACCAGAA ATGATTCGAC TCCCTTCCTC AGGTCGGGCC ACAAAAACGG CCCCCAAAGT
241 CCCTGGGACG TCTCCCAGGG TTGCGGCCGG GTGTTCAGAA CTCGTCAGTT CCACCACGGG
301 TCCGCCAGAT ACAGAGCTAG TTAGCTAACT AGTACAGACG CAGGCGCATA CTTTCNAAAA
361 AAATAGAA

1M-67 (left)

1 GCNACCTACA GGTGGGGTCT TTCAATGCTA GCTATCCAAT CACACCAGCT CTCTGCATTC
61 TTAACAGAGG GTCCTTTGTT CCAAACAGTA AGGCTCAAAA GAAACATAGT CTGATAAAAT
121 CACACTGAGA TCAGTACTCC CAGCTGGTGC AAGGGCTGAT GCCATTCCAT AACACACCCT
181 ACAGGAAGCA GCCTGAGATA CACAGCTGAT TGATTTCCAC AGCAGGAGCC TTGTGGCAGT
241 GTGGGGTGGG GACTGGTCAG GCATAGAGAA AATTCTTCAG TCGCTGACTC AGATTAGTTC
301 CCCAAGATCC TGACTCTATC TGTGTAAGTC ACTCCCGGAA GCAACACTAT TTCTTAACTA
361 ATTTTCTTAA AAGGAGAGGA AAACCACAGA AGCACCCCCA GCGAGTGGAA CTATTTAATC
421 AGTTAACTCT TACTTGTCAT GGGATCTGAA GATGGCAAAA ATCAAATTCC CTAGCTTTCT
481 AGAAGTAAGC TGAATAATTC TTTCATTGTA AGGGGCCGTC ATCCTGTGTA TTGTAGGTTG
541 TTTCGCAGCA CTTACTAGAT GCCAGTAGCA GCTTTTCCAG TCATAACAAT AAAAAATGTC
601 TCTGGGCCAG GCACTGTGGC TCATTCCTGT AATACCAGCA CTCTGAGAGG CTGAGGTAGG
661 CAGATCATTT GAGCTCAGGA GTTCAAGGCC GGCCTTGGCA ACATGGTGAA ACTCTATCTC
721 TACAAAAAAT ACAAAAATTA GCCAGGAATG

1M-67 (right)

1 GGGAGGGTCT CCTCTGAGTG ATTGACTACC CACGACGGGG GTCTTTCAGC ATATACATAC
61 ATGGTTCCTA TGGGAAAAAA ACAGACATAA ATTATGCTAT AGCCCTCTAA AATTTTCATC
121 TCCAAAATGA TTTCCTGACC TTCTGTCTGT ATTCACAGTT CTTTCTGTAA GTGCTTATCC
181 TTCCCCCAAA GCAATTCGAT AAAATAAAAG ATTTTATTTA GTCTCCAGAA AAAGGGGGGA
241 ATGAAAGACN CCNACCTGTA

1M-72 (right)

1 TNNGNTCTTG GGAGGGTCTC CTCTGANTGA TTGACTACCC ACGACGGGGG TCTGCATCCA
61 GAAGTCTAAA TTTGGATTCA CCTTATTGTC AGGGATGTGA AAGCCTCCCT TTCTCCCTCA
121 AAAACAAAAC AAGGATTGCT GTAATGCAGT ATTTTGCACA TAAACGTTCA AACCTTTATC
181 AAACAAAAAT CCCTCGCAGA ACAAGTAGAA TGGTATTAGA TGCACAGACA TCTTGGGAGT
241 TGGTGCAGAA ACATCAGTAG ACAAAACTGA TGGAATCATT TCCCACAAAG CAGGTTTGGC
301 TTAGGCTGGG GGCACTCTGT CAAATGATTA TTTTACCCAT TAACGTTTAA GCGGTTTTCT
361 TTAAATGAAA AACGTCTCAC AAAATCTGAA GTTACCATCA ACACTTTTAT CTTAAAACTC
421 ATAATCAACG GTATGAGTAA TTCCAACTGC CCACCAGCAC ACGGCTTATA AAATACCTTG
481 AAGTCAGGCC TCACTTGGGT AAGAAACCTG CCCTCCCTCT CCTCTCACCA GAGACTTTGC
541 CTTACTTACT TTGCCACAGA AAGAGCAAGT GTACTTGGCG TGCTGGCTGA TTTCAATTTT
601 CTTCACCATT TTCCGGAGGG AGGCCCCATA GCGGGTCCCG TATTTACCGA CGATCCCGAC
661 TTTCTTGGTA CGTTTGGCCT GTTTAGAGTG AAGGGAGAAA CCAGTGATAG ACCTGGNAAC
721 CCAGGCAGGC CCTGGAGCC

1M-73 (left)

1 CNGCTGCCAA CCTACAGGTG GGGTCTTTCA CAGTTTTTAC CTAAGGAAAA AAGTATAAGA
61 CAAGACATTT TAAAGTCAGA TTACTAAAAT GATTCCTGTA ATTCCACATT CTCAAGAAAA
121 AATGTGTTTA AGGTGTAATA GTAAATTCTT ATAGGACTTA TTCTAGTAAG TGGGTAAATG

181 GTAAAAATCT AAATTTGGGT TAAATTTTTT CGCTCAAATA CCTACATTTC CTTTATCGTA
241 CCCCAAAAGG AGCTCTCAAA GAGTAGTTTG AACAGTTTTT GACCCGAAGA TTTTTTTTTT
301 TTNNANNNGG TNGNACNCTN CGGNTNCCGG NANGGAAAAA AAAGGCCCTT NAGGGGNGGG
361 NNNACTNNTT TCAGGTNGGT NTTATTTNAA NGAAAAANNA ANGGNGTTTN GGGATTTTGT
421 TNNAAACNCN CANCCAAAAG GGTTTTNCNA CNCCCTNCCN CNGGNNGGGC CANAAAAANG
481 GCCCCNAAAG CCCNGGGNAN NNCCCCCNGG GTNGNGNCNG GGGGTNNAAA ACNCGNCANT
541 NCCCCCNNGG GCCCCCCAAC GNAANNNTTT TTNTTNGAAA

1M-73 (right)

1 GGGAGGGTCT CCTCTGAGTG ATTGACTACC CACGACGGGG GTCTTTCAAC TGTATATCTG
61 CTGTATGGCT ATGGAGACTC AAAATCCTCA GATTTGGTTA CTTACATTAG CCAGTTTTTG
121 GTTGTGTAGA TTATAACAAG GAAATACCCC GTTTCTAAAT ATAGAAATTG AAAGAATTTT
181 TGTATGACCA GTTAAATTTC AAAAATAGCT ACGTAGAGTG CACACCAATT TCTTAAGGGC
241 CCATATGTTT GAATATTTTA AACAAGATTT ATACATTAGA TGGTCCAAAC AATTGCTTTA
301 ATTCCTCACA CTTCTCATGT GTAAATACAG AATTTAGAGA AGTCACCATG TTACTTTCAT
361 TTTCAATCCT GCAAATGTGT CAGTGTACAC TTATGTAAGG ATTCTTTGGT TTTGACACAT
421 TCATATCCTA GAAGACATAA ATATTTTTAC TAATGCAGAC TTTTGACAAC CTTATTCATA
481 TGAAAATGTA GCTTTTCCAT TTTCACCTTC TAAAGTTTTG TCCTCCCACA CAGAGCTAAG
541 TGGAAAATAG TAACTGCTAA CTACATTCGG ACTACATAAA ACATCATCTG AGTTGTGGGC
601 CTGAATTACC TAGGTATTTG CCACTTCACA TTTACATTTG TGTAAATATG ACTAGAATGC
661 CAGGTCAAAT ACCTTGTTTG GTAAAGCACA TTACAATTTG ACTCAATGAA TAGCTATGGA
721 AGTTTTCTTT ATTGGATTAC TTAATGTGTA ACAATAAT

1M-75 (left)

1 NGCTTGCCAA CCTACAGGTG GGGTCTTTCA GGTTAAAGGG CAGAGGAGAG AGCTCTGGAC
61 ATGGTGGCTG GAAGGAGGCC TGGATTTGAA TCCTGGCTGT TTGGGACCCT GGAGGGGTCA
121 GTCATCTGGG AGTTTTGAGT CCTGGATTCC CAAGGGACCC TCAAGTTCTC TTTTCCCCAG
181 AGCAGTACAG GGGACCCAGC CCATGAAGAC CTCGGTTTCC TTCTGATGGA GAGGTGGTTC
241 CTGTATAGGA GGCTCCAGCG TGTGTGGTCA GTCAAGTTCA AGGCCCCTGT GCTTTTGGCC
301 TTGGGCCAGT GCAGGACTTG ACTGCACTGT GCCTCACTCA CTGTGTAAAA TGGATCTAAT
361 TATGGGATCA CTGTGGCGAG AAAGGAAAAA TATAGGTAAA ATCCTTGCAC AGAGCCTGGC
421 ATGAGAGGGA AAGGGGCTCG ACTCCCTTCC TCAGGTCGGG CCACAAAAAC GGCCCCCAAA
481 GTCCCTGGGA CGTCTCCCAG GGTTGCGGCC GGGTGTTCAG AACTCGTCAG TTCCACCACG
541 GGTCCGCCAA NGAAANNTNT TTNNTTAAAA

1M-75 (right)

1 TGGGAGGGTC TCCTCTGAGT GATTGACTAC CCACGACGGG GGTCTTTCAT AACCCTTTGA
61 TGCTCTTTAA ACCCTCACAT GTCCACATGA CACTTTCGCA TCCGTTCTCT CCTGCTTAGC
121 CTCAACATCC TCAGAGAGAT GTTCGGGGCT TATCTCACCA TGTCCATTGC ATAGAAGGGG
181 AAACTGAGGC CTGGAGCGTG TGTGGGCCAC CAGATCTCCC CCTGACCCAC AAGGGAGTGA
241 GTGATATTTG CTTTGGTGGA GGATAAATTT GGAGGCTGGC CTCTACCCCC GGCCCTGCCC
301 CTGGGAGAAG CCTACCGTGG TCTGTGCCTG CACACGCCGC TCCGGATCTG GAAGGTTTGC
361 CAGGTCTTCA GTGGGACACC ATCGTCTTCC GGGCCAGGCC CCCTCTGCTT CCCTCTCCTG
421 CTGGATCTTC TCTGGAGTCG GCTCCTCACC CCGCCCCCAA CTTGACTTCC CGTCCAGAGC
481 TGGGTGGCCC TGACGCTACC CCCACTTCCA CCCCCCACCT CCAGTTGAAG GGCTGGGGGC
541 AGTAGGTGCA GAGGGCCCTG GGTTCCCACC CAGTCTGTGC CTGACACAGG TTGGGGCAGG
601 GTCTTGGGAG CCAAGGGGAG AAGCAGGGCC GGGGTGTGGG GAGGGGGCGT CCTTGTGCCC
661 GATGCCCCTG CCCAGTGCTG CGTGGCGAGG CCGCTGGGGC CTGAGTCACA GCTGACGGGG
721 CCGCGCCCCG CCTGCCT

1M-83 (left)

1 NGCTTGCCAA CCTACAGGTG GGGTCTTTCA GAAGTGGATG AGGCGGGCCG CGGAAGCGGG
61 TGAAAATGGC CTGCGGGGAG CTGTCGTCCA GGCTGAGGGT CTTCCAGGCG CCTGACTCCA
121 ACCCCGAGTG ACTCCAGCCC CGGGCAAATG GGACTCGGAC TGCTTTGCCG CAGACTCAAA
181 ACTTGGGCGC CTGGGCTGAG CTCAGCACCG CCCCCTGGTG CCCAGTCGGG CTCTGCGGTG
241 CCGCCGTCTT GGGTTCCCCG GACATCTGTC CAGTGGTCGA CTCCCTTCCT CAGGTCGGGC
301 CACAAAAACG GCCCCCAAAG TCCCTGGGAC GTCTCCCAGG GTTGCGGCCG GGTGTTCAGA
361 ACTCGTCAGT TCCACCACGG GTCCGCCAGA AANAGANCTA AGTTTAGAA

1M-83 (right)

1 GGTCTCCTCT GAGTGATTGA CTACCCGTCA GCGGGGGCAT TTGGGGGCTC GTCCGGGATC
61 GGGAGACCCC TGCCCAGGGA CCACCGACCC ACCACCGGGA GGTAAGCTGG CTGCCTCGCG
121 CGTTTCGGTG ATGACGGTGA AAACCTCTGA CACATGCAGC TCCCGGAGAC GGTCACAGCT
181 TGTCTGTAAG CGGATGCCGG GAGCAGACAA GCCCGTCAGG GCGCGTCAGC GGGTGTTGGC
241 GGGTGTCGGG GCGCAGCCAT GACCCAGTCA CGTAGCGATA GCGGAGTGTA CTGATAACTT
301 CGTATAATGT ATGCTATACG AAGTTATTAG GTCTGAAGAG GAGTTTACGT CCAGGAGCGC
361 ACCATCTCCT TCAAGGACGA CGGCAACTAC AAGACCCGCG CCGAGGTGAA GTTCGATAAA
421 ATAAAAGATT TTATTTAGTC TCCAGAAAAA GGGGGGAATG AAGACCCCCC CCCCTTGTAA
481 A

1M-84 (left)

1 GNAACCTACA GGTGGGGTCT TTCACAGAAC TAGGCAGGGT GGGTAGGGGA AGACCTCAAG
61 ACAATGGAGA GTGGGAAAAT AGACGCATTT AATATTAGAA GCATCAGAAA AAAAGGAAAA
121 CAACACATTT TTTCCCCCTA GAACTACACA GGGAGGTGGG GGAGGAAGAT GGGGAAATAG
181 AAGGAGAAAC TGACTAAGGA CCTTGTTTGG GGGAGATAAA AGGTATAAAG GACACAGAGG
241 TAAAGACGTT CCGGAAAGCT CTAGAGGAGC CGCCCTCTCC TGTCTCGTCA CCATTTTTAA
301 AGGGATCTGA TCCTCGCAAT AGCTCTTGGT ATATGCTGGA GGTAATTGCC TAGATTTCCC
361 GCGTTCCTGT CCTCTGCGAG TCTCTGGCTT GTGACAGCCA CTGTGACTCT TCTGATGCAC
421 TTGTCAGCTA GAGGACCGCG TGAGATGCAA ACACAAAATC AAGGGGCGCC TAATGTCTGA
481 CGAGGCGCAT TCCTGGGCGC AGTGGGAGCC TCGACTCCCT TCCTCAGGTC GGGCCACAAA
541 AACGGCCCCC AAAGTCCCTG GGACGTCTCC CAGGGTTGCG GCCGGGTGTT CAGAACTCGT
601 CAGTTCCACC ACGGGTCCGC CAGACGGAGN GNNNTGGTTA AGAA

1M-84 (right)
1 GGAGGGTCTC CTCTGAGTGA TTGACTACCC ACGACGGGGG TCTTTCACTG TCACGTCCCT
61 TCTTTCCCTG TATCACAACA ATGTTCTCCC TTTCTCACCT TTGTTTCTCT TCATACTTGT
121 TTCTCTCTCT GTGTCTCTCT CATTCTCTTC CCTTCCAAAT GTAGTATTTG GAGACTGTAA
181 TATCGATAAA ATAAAAGATT TTATTTAGTC TCCAGAAAAA GGGGGGAATG AAAGANCCCC
241 CACCTGTANN

1M-89 (left)
1 NGCTGCCAAC CTACAGGTGG GGTCTTTCAC CAGNTACTTT GGGAGGCCGA GGCAGGTGGA
61 TCACAAGGTC AGGAGTTCGA CTCCCTTCCT CAGGTCGGGC CACAAAAACG GCCCCCAAAG
121 TCCCTGGGAC GTCTCCCAGG GTTGCGGCCG GGTGTTCAGA ACTCGTCAGT TCCACCACGG
181 GTCCGCCAGA TACAGAGCTA GTTAGCTAAC TAGTACAGAC GCAGGCGCAT AATNNCAAAC
241 ATAGA

1M-89 (right)
1 GGCTNTCTTG GGAGGGTCTC CTCTGANTGA TTGNACTACC CACGACGGGG GTCTTTCACT
61 GGGATTACAA GTGTGAGCCA CGCGCCCAGC CCACCTGGCT AATTTTTGTA TTTTTCATAC
121 AGACGGGGTT TCACCATGTT GGCCGGCTGG TCTCGATAAA ATAAAAGATT TTATTTAGTC
181 TCCAGAAAAA GGGGGGAATG AAAGACCCCA CCTGTAGGTT TGGCAAGCTA GCTTAAGTAA
241 CGCCATTTTG CAAGGCAAGN AA

**Figure 2**. Sequences of *DHFR** integration sites determined from inverse PCR products. Primary inverse PCR products (0.1-1 µl) were used as a template in nested PCR under the same conditions, except secondary primers were used. The secondary primers used were Taq3, Taq3' (left PCR) and Taq 4 and Taq 4' (mixed PCR). Secondary PCR products were separated on a 1% agarose gel, purified using the QIAquick Gel Extraction Kit (Qiagen) and sequenced (sequencing primers Taq 3' and Taq 4). Sequences always contain part of the LTR sequence (yellow) followed by unique flanking genome sequence (green). Additionally, they sometimes contain the Taq$_{\alpha}$ I restriction site (black) and vector sequence (yellow). Sequences were mapped on the human genome sequence using BLAT software (UCSC Genome Browser, May 2004 freeze).

Primer sequences:
Taq 1: CTA TGT TTG ATG TTA TGC GCC TGC
Taq 2: CTA ACT AGC TCT GTA TCT GGC GGA
Taq 3: AGA GAA CCA TCA GAT GTT TCC AGG
Taq 3': GCC TTG CAA AAT GGC GTT ACT T
Taq 4: AGT TGC ATC CGA CTT GTG GTC
Taq 4': ACA GGT GGG GTC TTT CAT TCC
