# Supplementary material for: Genome position and gene amplification
Source: Genome Biol. 2007 Jun 21;8(6):R120. doi: 10.1186/gb-2007-8-6-r120 (PMC2394771; doi:10.1186/gb-2007-8-6-r120)

Additional data file 6. Comparison of copy number ratio data with and without spatial and GC content correction.
Spatial and GC-content corrections are used to reduce the noise in the copy number profiles; however the corrected copy number profiles do not differ in their copy number alterations.

(**A**) Procedure for geometric correction. The goal of the geometric correction method is to iteratively obtain an estimate of the systematic spatial variation of the log2ratios over the array. This estimate is then subtracted from the original raw ratios. An example comparing the data analysis for the elucidation of copy number alterations specific to drug resistant colonies is given for the 1M-84 insertion site using the original array data without any correction and with correction. The profiles without correction show slightly greater ratio variation, but no differences in copy number alterations.

(**B**) The linear regression plots and copy number profiles of resistant colonies from the 1M-84 insertion site are shown for each of the four colonies without (left panels) and with (right panels) geometric and GC correction.

(**C**) Whole genome copy number plots for methotrexate resistant colonies without (left) and with (right) spatial and GC content correction. The red lines indicate the segments as determined by applying the circular binary segmentation (CBS) method. The blue lines indicate the results of applying the mergeLevels procedure to segments which were not statistically different in their distribution. Green dots indicate amplicons and yellow dots, the clones which were outliers in a segment.

(**D**) Heatmaps of the copy number alterations specific to methotrexate resistant colonies as determined using hybridization ratios without (left) and with (right) spatial and GC content correction.

A. Procedure for Geometric Correction

1. Order the UCSF SPOT log2 ratio data by genomic position
2. Apply a sliding window (one-dimensional) median filter, to estimate extended genomic features
3. Subtract the estimate in step 2 from the data in step 1, yielding "flattened" data
4. Arrange the flattened data back to their original two-dimensional array positions
5. Apply a (two-dimensional) sliding window median filter (default size 3x3) over the data in step 4. This result is our initial estimate of the systematic geometric variation in log2ratios over the array.
6. Subtract the result from step 5 from the original raw UCSF SPOT log2ratios, to produce the first version of the "corrected" UCSF SPOT log2ratios.
7. Using the result from step 6 as input to step 1, repeat steps 1 through 6.

In our experience, three iterations of the above procedure are sufficient for the data at step 6 to converge to a stable result, which is taken to be the "corrected" UCSF SPOT log2ratios.

B. Linear regression and copy number profiles
1M-84_6

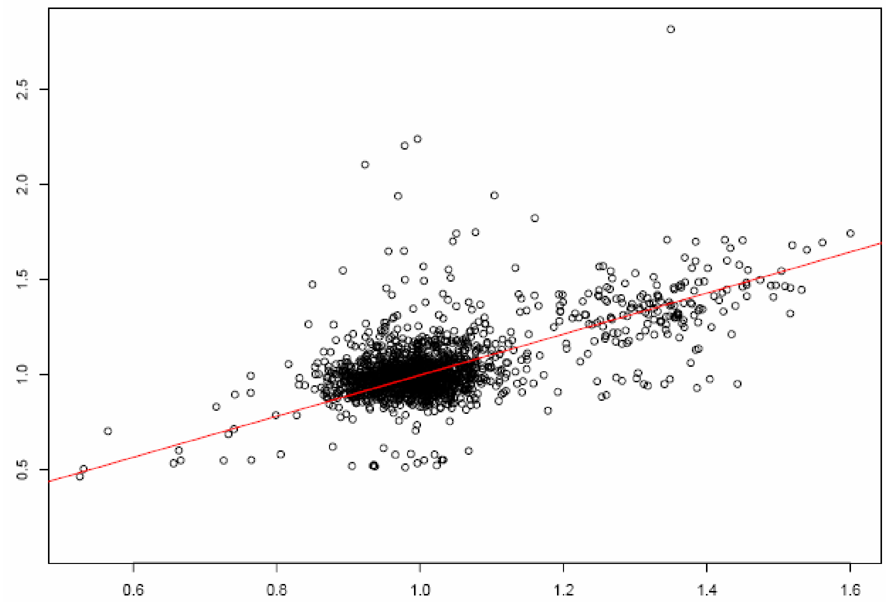

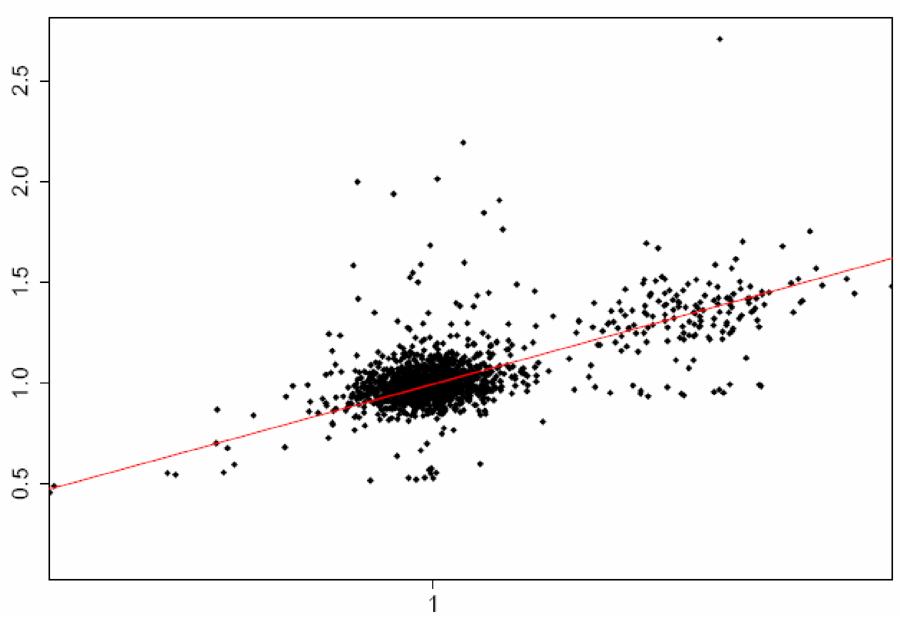

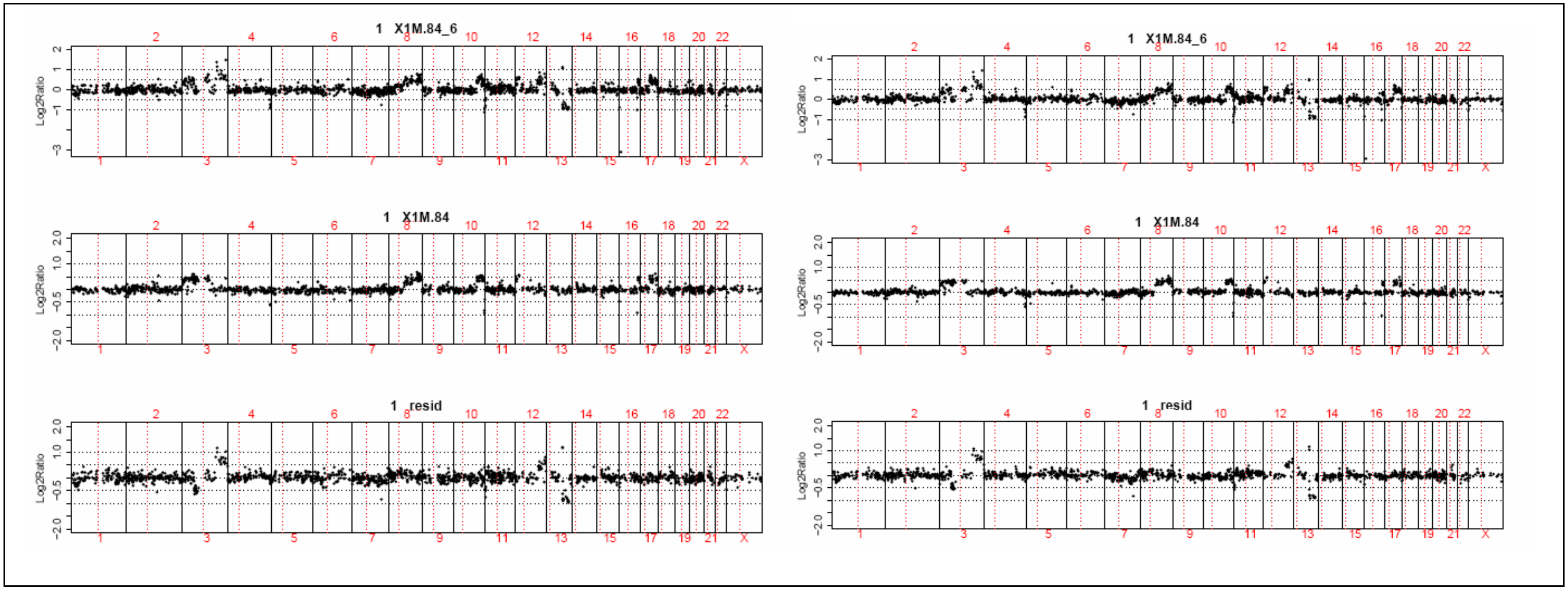

1M-84_8a

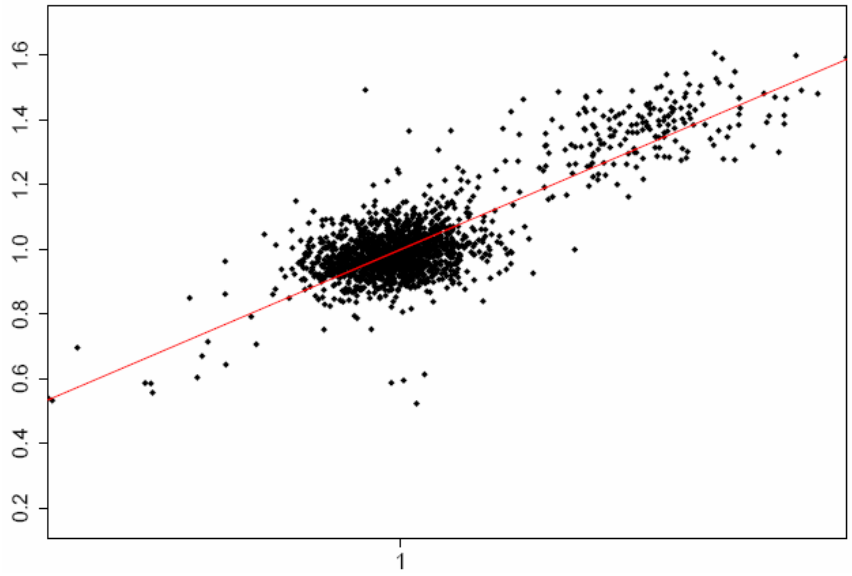
1.6
1.4
1.2
1.0
0.8
0.6
0.4
0.2
1

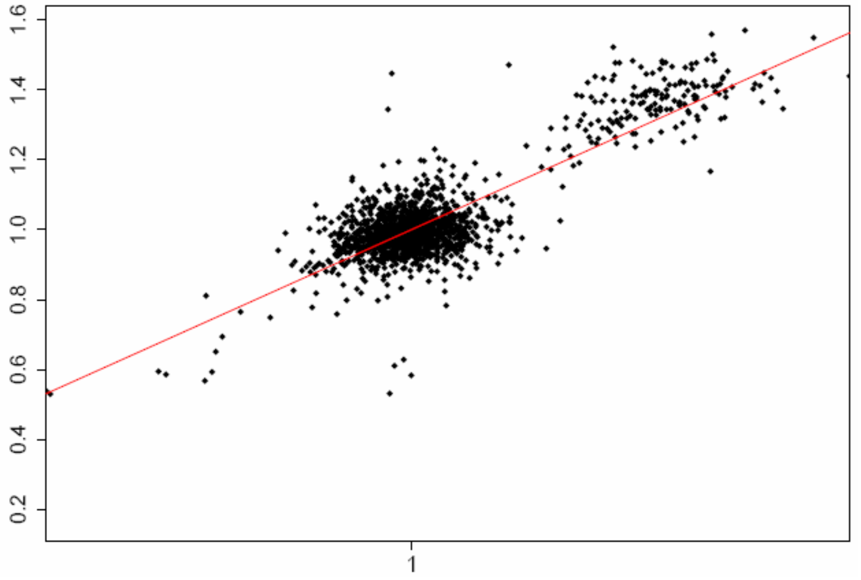
1.6
1.4
1.2
1.0
0.8
0.6
0.4
0.2
1

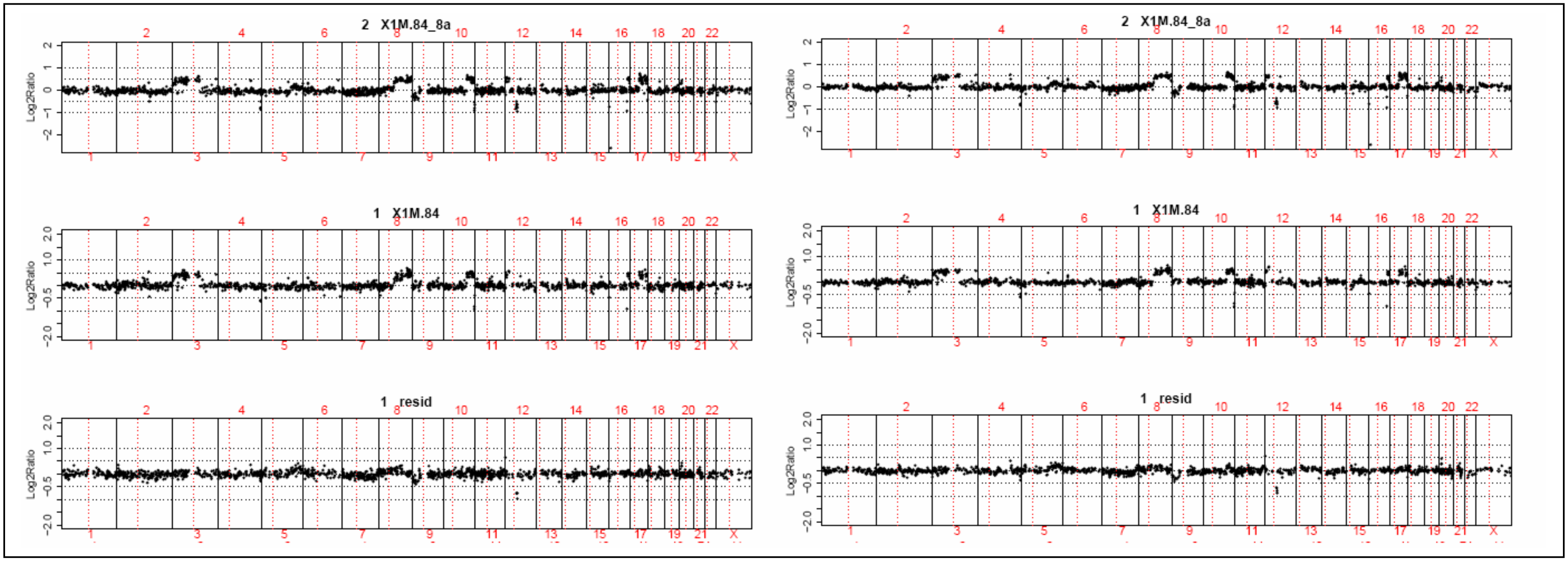
2 X1M.84_8a
1 X1M.84
1 resid
2 X1M.84_8a
1 X1M.84
1 resid
Log2Ratio

1M-84_c1

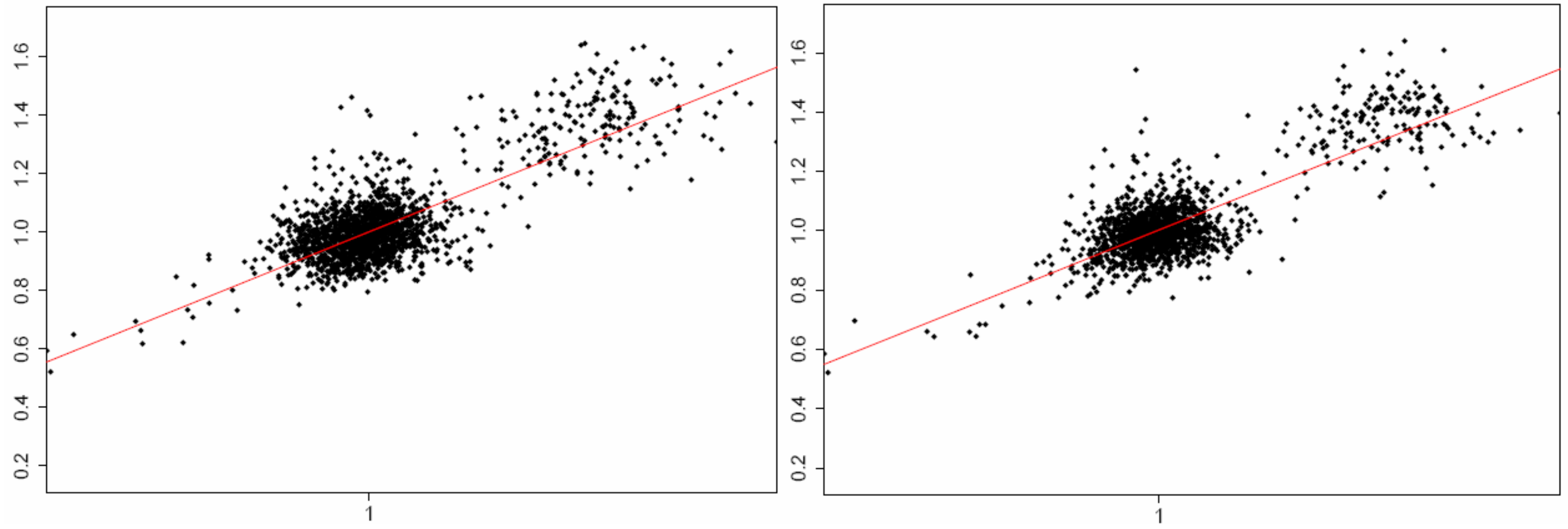

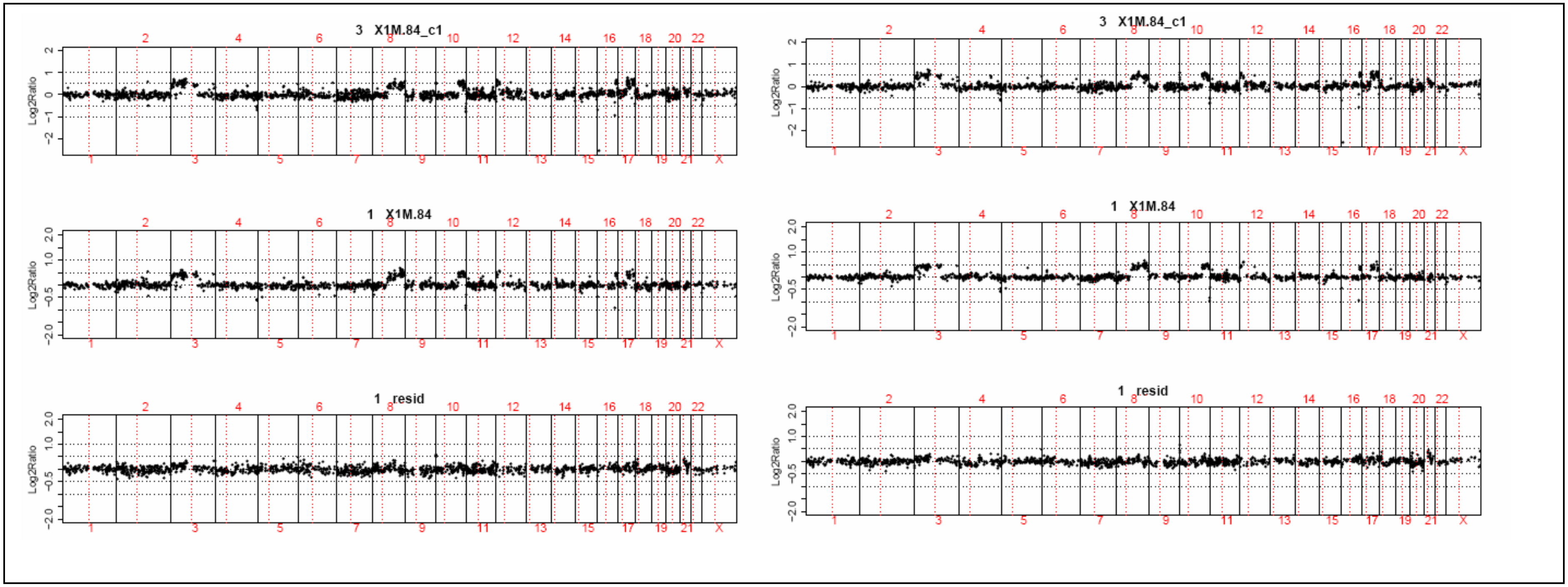

1M-84_c11a

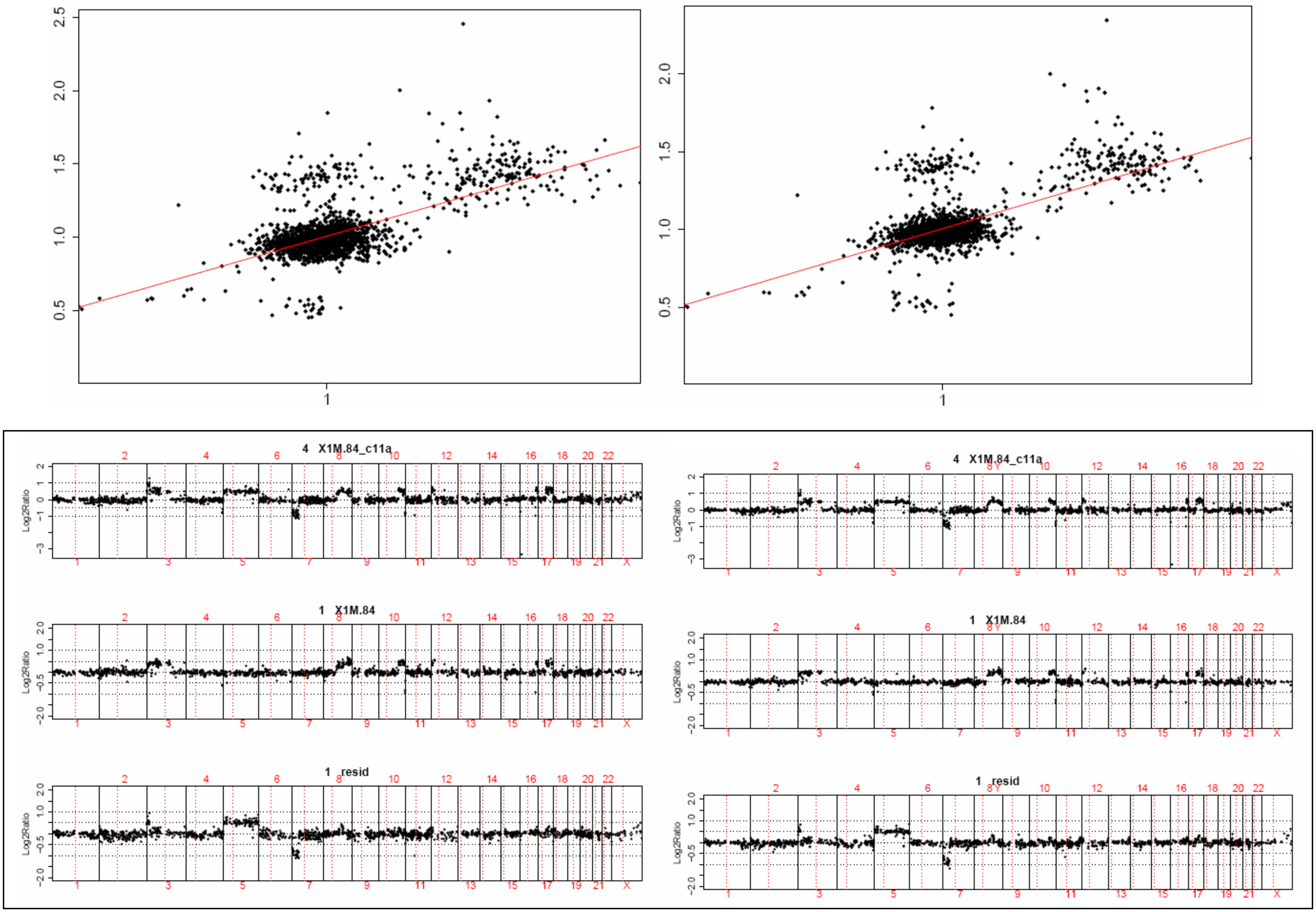

C. Genome Plots

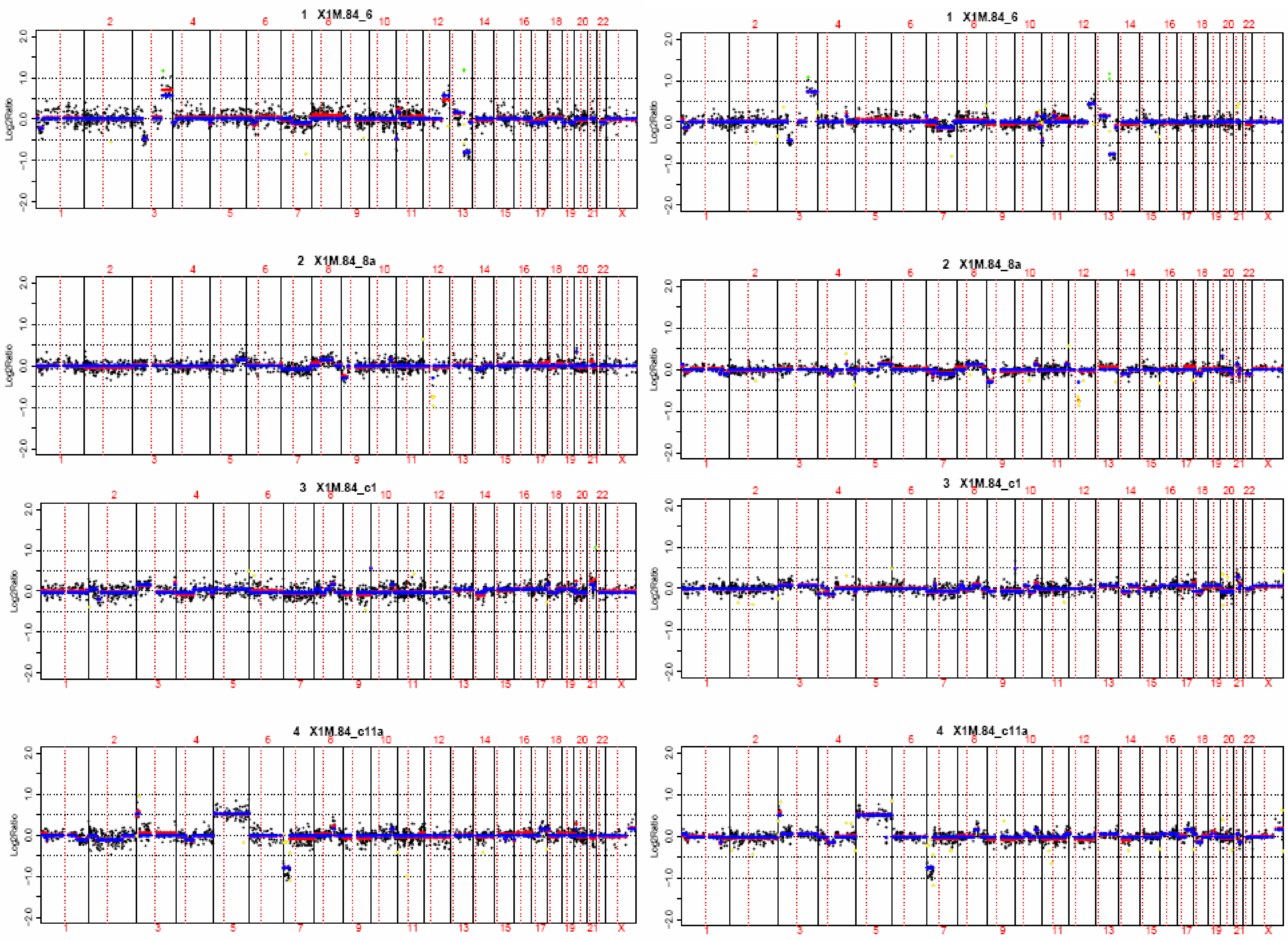

D. Heatmaps showing copy number gains (green) and losses (red)

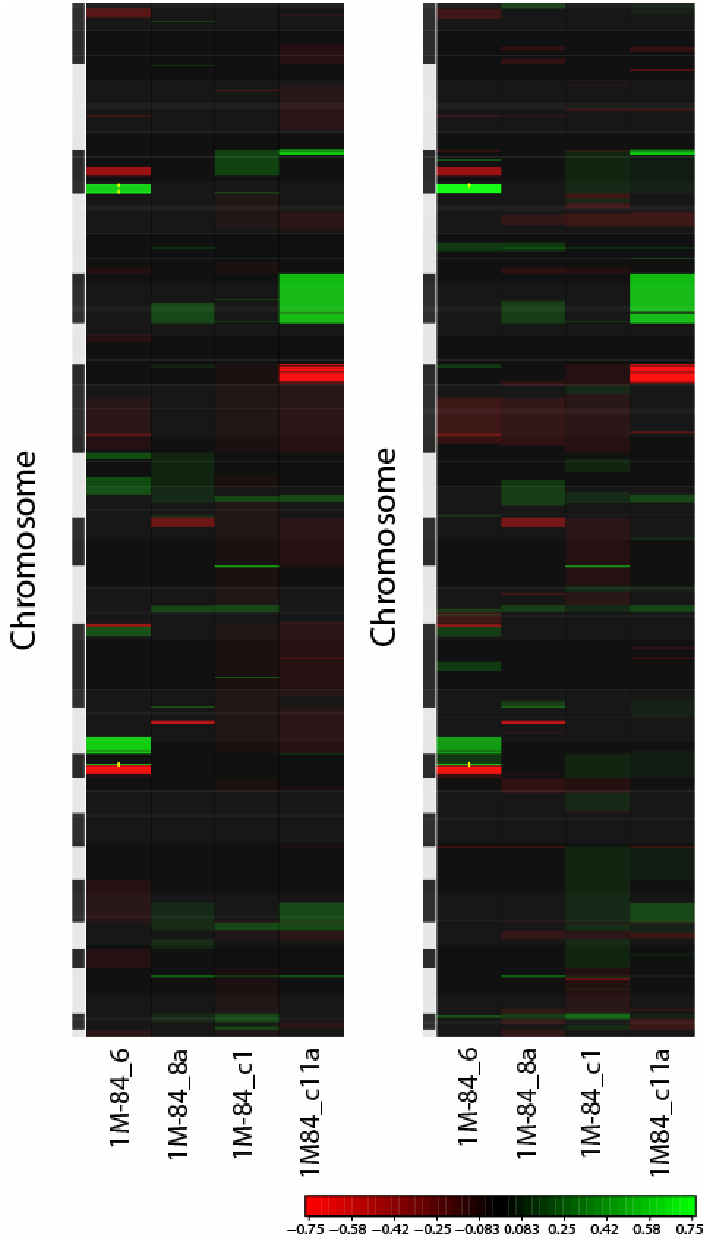

Supplement: Additional data file 6 — Comparison of analysis of array data with and without spatial and GC correction [file gb-2007-8-6-r120-S6.pdf]
