# Supplementary material for: Genome position and gene amplification
Source: Genome Biol. 2007 Jun 21;8(6):R120. doi: 10.1186/gb-2007-8-6-r120 (PMC2394771; doi:10.1186/gb-2007-8-6-r120)
Supplement: Additional data file 7 — Example of elucidation of copy number changes specific to methotrexate resistant cells (results of the analyses for the 1M-34_c5a colony) [file gb-2007-8-6-r120-S7.pdf]

Additional data file 7
Elucidation of copy number changes specific to methotrexate resistant cells

Figure 1.

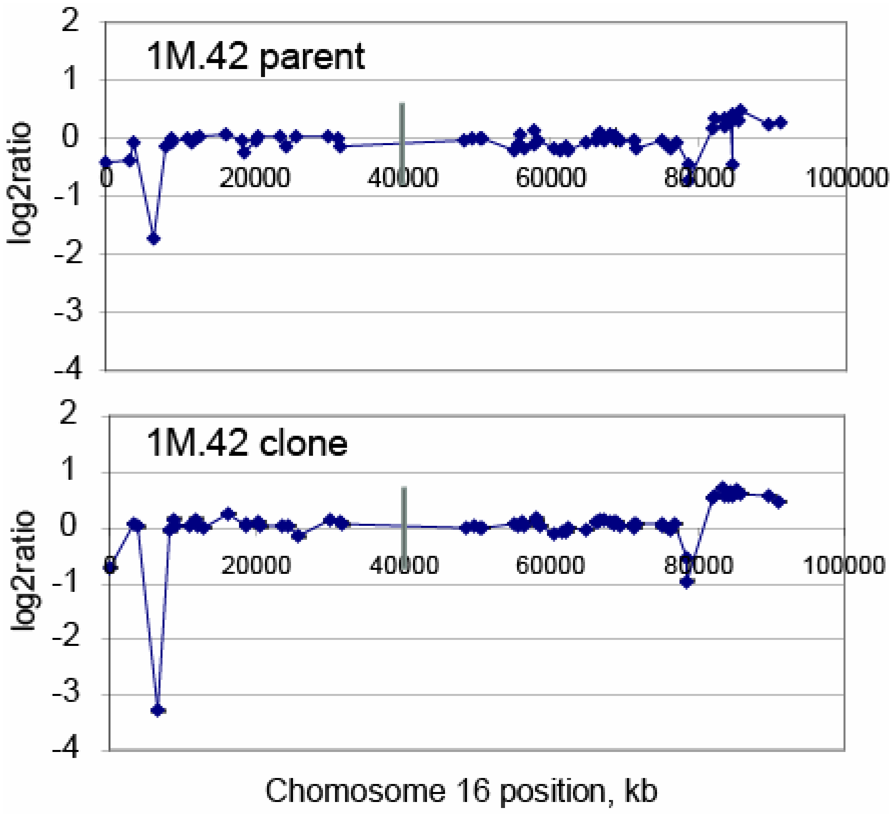

Figure 1. Copy number profile on chromosome 16 for the untreated 1M-42 parent and one of its methotrexate resistant colonies. The amplitude of the homozygous deletion on 16p and the heterozygous deletion on 16q is less in the parent than in the resistant colony.

Figure 2.

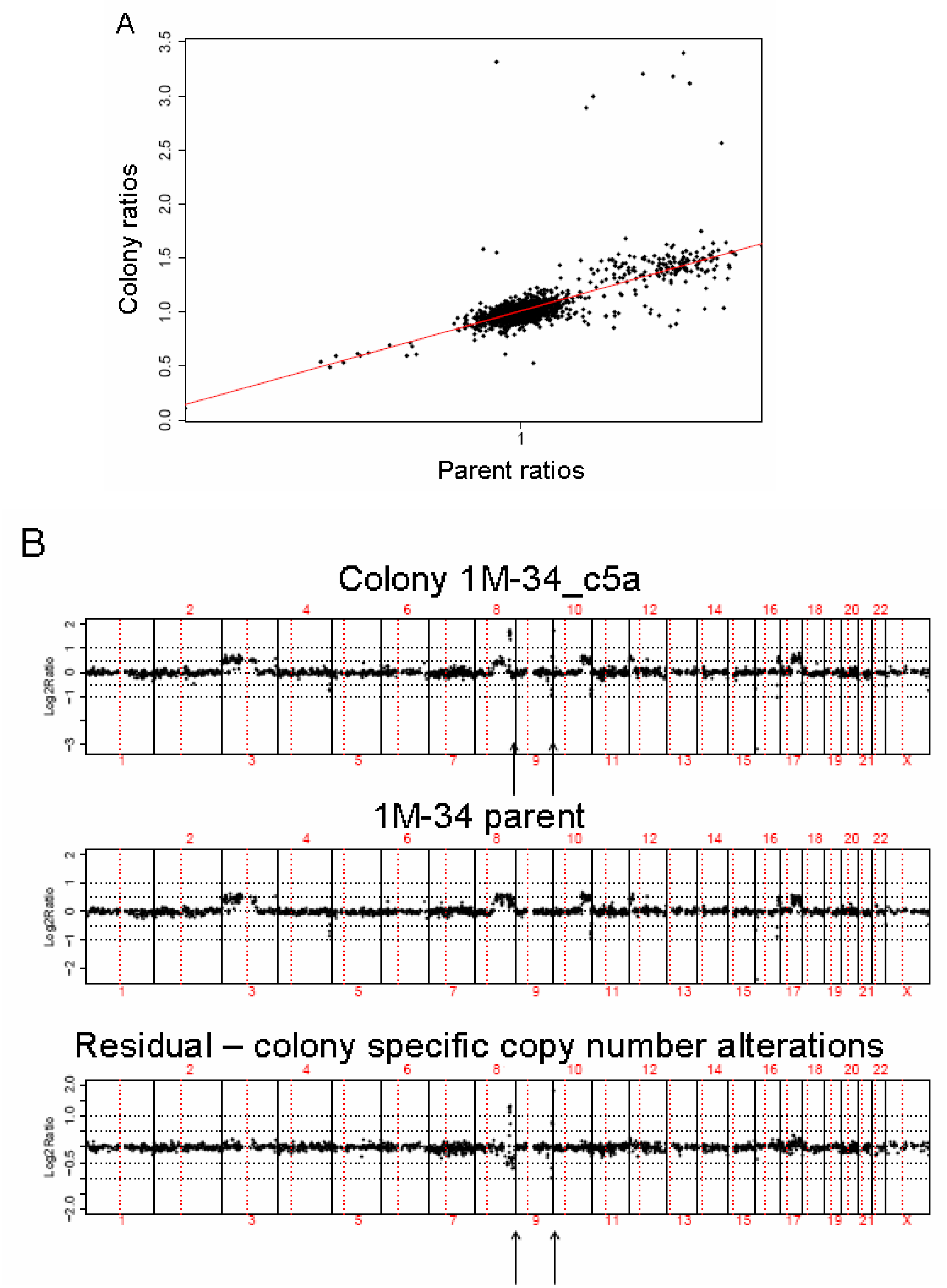

Figure 2. Elucidation of copy number changes specific to methotrexate resistant cells. (**A**) Determination of the fitted value for correction of differences in the amplitude of the copy number profiles of the 1M-34 parent and colony, 1M-34_c5. (**B**) Whole genome copy number profiles of the methotrexate resistant colony 1M-34_c5a, the 1M-34 parent (no methotrexate treatment) and the 1M34_c5a methotrexate resistant colony specific copy number changes after subtraction. Regions of colony specific amplification on 8q and 9q are indicated by the arrows in the resistant colony profile prior to subtraction and after subtracting the aberrations present in the parent. The original 1M-34_c5a chromosome 9 and 8 copy number profiles showing these amplicons are shown in Figure 3A and B, respectively. The analysis of all resistant colonies is provided in Additional data file 8.
